# Genomic evolution of enteric pathogens: mechanisms of pathogenicity and diagnostic innovations

**DOI:** 10.3389/fmicb.2025.1647437

**Published:** 2025-11-19

**Authors:** Tao Li, Jin Li, Zhiping Tang, Xing Liu, Shiwen Yao, Jiabao Zhu, Wei Wang, Linju Huo, Song Chen, Gaihua Zhang, Zhonghua Liu

**Affiliations:** The National & Local Joint Engineering Laboratory of Animal Peptide Drug Development, College of Life Sciences, Hunan Normal University, Changsha, Hunan, China

**Keywords:** pathogenic bacteria, genome evolution, rapid diagnostic methods, pathogenicity mechanisms, gut microbiota

## Abstract

Genomic evolution serves as a pivotal driver of pathogenicity and host adaptation in intestinal pathogens. This review systematically dissects, from a phylogenetic perspective, the key genomic evolutionary mechanisms underpinning pathogenesis across five major classes of intestinal pathogens and their significance. Bacteria (e.g., *Escherichia coli*) acquire virulence- and antibiotic resistance-enhancing genes via horizontal gene transfer and genomic recombination, equipping them to disrupt the intestinal mucosal barrier and evade host immune defenses. Fungi (e.g., *Candida albicans* and *Cryptococcus* spp.) significantly augment their pathogenic potential through chromosomal rearrangements and dynamic expansions or losses within gene families. Parasites (e.g., Giardia lamblia) successfully evade host immune recognition and clearance through complex life cycles and stage-specific gene expression regulation. Viruses (e.g., rotaviruses and noroviruses) rapidly adapt to host cellular environments via genomic mutation and recombination, triggering acute gastroenteritis. Although prions primarily propagate via the nervous system, the pronounced cellular stress response they elicit in intestinal tissues suggests the gut may serve as a potential secondary transmission or amplification site. Collectively, these diverse evolutionary mechanisms confer unique colonization, survival, and competitive advantages upon distinct pathogen classes within the complex gut microenvironment. Employing *Escherichia coli* as a paradigm, systematic bioinformatic analysis of 335 key virulence factors revealed evolutionarily stable functional clusters (e.g., effector/toxin systems, 21.0%) with core contributions to pathogenicity. These conserved genomic signatures provide a robust foundation for developing novel high-precision diagnostics. For instance, CRISPR-based platforms achieve 100% clinical concordance in detecting the Shiga toxin gene (stx2), while loop-mediated isothermal amplification coupled with lateral flow assay (LAMP-LFA) enables rapid (< 40 min) and accurate detection of *bla*_NDM − 1_-mediated carbapenem resistance. The deep integration of multi-omics data (genomics, transcriptomics, proteomics, etc.) with artificial intelligence (AI) is substantially accelerating the discovery of novel biomarkers. Looking forward, innovative technologies such as real-time nanopore sequencing and nanomaterial-enhanced high-sensitivity biosensors hold promise for achieving rapid, broad-spectrum pathogen detection, thereby robustly supporting the World Health Organization (WHO)'s “One Health” strategic goals. In conclusion, the “Genomic Evolution–Biomarker Discovery–Diagnostic Development” integrated triad framework presented herein offers crucial insights and actionable pathways for advancing next-generation precision diagnostics and formulating effective global infection control strategies.

## 1 Introduction

The human intestine, as the largest immune and metabolic organ, harbors a complex and diverse microbial ecosystem essential for maintaining host health. According to the Global Burden of Disease Study, diarrheal diseases were responsible for an estimated 1.17 million deaths worldwide in 2021 [95% uncertainty interval (UI): 0.793–1.62 million] and accounted for 59 million disability-adjusted life years (DALYs), with viral pathogens such as *rotavirus* constituting major contributors. Although diarrheal diseases represent a global health threat, their epidemiological burden varies substantially across regions and age groups. Low- and middle-income countries (LMICs), particularly in sub-Saharan Africa, experience the highest burden, with diarrhea-related mortality reaching 151.9 per 100,000 population among children under 5 years of age. In contrast, high-income countries are primarily characterized by increased hospitalization rates and healthcare expenditures among elderly and immunocompromised populations, as exemplified by *Clostridioides difficile* infections (Collaborators, 2025).

The pathogen- and demographic-specific burden of diarrheal diseases further underscores their complexity. Among bacterial pathogens, *Shigella* is estimated to cause ~81,800 deaths annually in children under five, contributing to 7.34 million DALYs and ranking as the second leading cause of death in this age group. *Clostridioides difficile* predominantly affects individuals aged ≥70 years, resulting in ~15,600 deaths and 284,000 DALYs, often in the context of antimicrobial misuse. Enterotoxigenic *Escherichia coli* (ST-ETEC) and typical enteropathogenic *Escherichia coli* (tEPEC) exhibit high population attributable fractions (PAFs) of 13.4% and 13.6%, respectively, highlighting their relevance in pediatric populations. Among parasitic agents, *Cryptosporidium* is responsible for an estimated 118,000 deaths and 7.37 million DALYs globally, with a PAF of 20.1% among children under five. *Entamoeba histolytica* exceeds a 5% PAF in this age group, although its precise global burden remains uncertain. Viral pathogens remain predominant contributors to disease burden; *rotavirus* alone accounts for ~176,000 deaths annually—of which 120,000 occur in children under five—and 10.8 million DALYs. Additionally, *adenovirus* and *norovirus* are associated with ~81,100 and 124,000 deaths, respectively (Collaborators, 2025).

Fungal pathogens constitute a substantial burden on hospitalized and immunocompromised populations. As reported by *The Lancet Infectious Diseases* (2024), invasive fungal infections result in an estimated 6.5 million cases and 3.8 million deaths annually, with 68% of fatalities attributable to fungal etiologies. *Candida* species cause ~1.565 million invasive infections and 995,000 deaths per year. *Cryptococcus* meningitis accounts for ~194,000 cases and 147,000 deaths, primarily affecting HIV-infected individuals in sub-Saharan Africa. Infections caused by *Aspergillus* species are estimated to exceed 2 million cases and 1.8 million deaths annually. The burden of endemic fungi, such as *Histoplasma*, is thought to affect ~100,000 individuals globally, although data remain limited (Vallabhaneni et al., 2016).

Although prion diseases (e.g., *Kuru*, variant *Creutzfeldt–Jakob* disease) can be transmitted via the oral route, they are rare and are not generally categorized as enteric pathogens. Experimental models suggest that intestinal inflammation may enhance prion transmissibility; however, global burden estimates are currently lacking.

Sub-Saharan Africa continues to be the region most severely affected, with an estimated 14 million DALYs attributable to 85 pathogens, accounting for 61.5% of the total regional disease burden. This reflects the high prevalence of coinfections involving *rotavirus, Plasmodium* (malaria), and HIV. In South Asia, HIV and *rotavirus* are major contributors, jointly accounting for over 10 million DALYs among children under five. These data underscore the urgent need for expanded vaccination coverage and targeted public health interventions. Even in high socio-demographic index (SDI) settings, enteric pathogens remain a significant concern. For instance, surveillance in Zealand, Denmark has shown that *rotavirus* and enteropathogenic *E. coli* (EPEC) predominate in pediatric winter infections, whereas bacterial pathogens are more frequent in elderly patients during summer. These epidemiological trends highlight the necessity of implementing region- and age-specific precision public health strategies (Group, 2024; Johansen et al., 2023).

In addition to epidemiological determinants, genomic evolution plays a critical role in shaping virulence, antimicrobial resistance (AMR), and host adaptation in enteric pathogens. Mechanisms such as mutation, horizontal gene transfer (HGT), and chromosomal rearrangements collectively drive these processes. In bacterial pathogens, the diversification of virulence factors and resistance determinants underlies their pathogenic potential and informs biomarker discovery. Understanding these evolutionary mechanisms is essential for elucidating microbial biology, monitoring evolutionary trajectories, and guiding the development of next-generation diagnostic tools (Scott et al., 2025; Quan et al., 2023).

This review proposes a closed-loop conceptual framework, designated the Genomic Evolution–Biomarker Discovery–Diagnostic Development framework, which integrates microbial evolutionary dynamics with translational diagnostic strategies. It systematically connects pathogen genome evolution to the identification of robust molecular biomarkers and the advancement of rapid, accurate diagnostic platforms.

The manuscript is structured into three major sections:

(1) **Evolutionary Mechanisms Across Enteric Pathogens**: A comprehensive overview of key evolutionary processes—including HGT, chromosomal rearrangements, hypermutation, and epigenetic regulation—in bacteria, fungi, parasites, viruses, and prions, with emphasis on their roles in virulence, AMR, and host adaptation. Table 1 provides a systematic summary of the evolutionary mechanisms of the five enteric pathogen classes for clear and intuitive reference.
(2) ***Escherichia coli*** **as a Model**: Functional annotation and classification of 355 *E. coli* virulence genes to illustrate evolutionary patterns and demonstrate how genomic insights guide biomarker discovery and pathogen profiling.
(3) **Molecular Diagnostics Guided by Evolutionary Biomarkers**: Discussion of diagnostic platforms, including CRISPR-Cas and loop-mediated isothermal amplification with lateral flow assays (LAMP-LFA), emphasizing their application in detecting conserved virulence and resistance markers across diverse pathogens.

**Table 1:** Hierarchical summary of genomic evolution mechanisms driving pathogenicity in intestinal microbes.

**Primary microbial group**	**Subcategory of evolutionary mechanisms**	**Specific evolutionary mechanism**
Bacteria	Horizontal gene transfer (HGT)	Conjugation
		Transduction
		Transformation
	Antibiotic resistance	Intrinsic resistance
		Acquired resistance
		Adaptive resistance
	Virulence gene recombination	Phenotypic plasticity regulation
		Coordinated regulation of molecular system transcription networks
		Mediated precise regulation
Fungi	Chromosomal rearrangement	Aneuploidy formation
		Chromosomal translocation and fusion
		Chromosomal misplacement
		Homologous recombination and gene rearrangement
	Gene family expansion/loss	Gene loss
		Gene amplification
	Evolution of drug resistance	Target gene mutations
		Upregulation of drug efflux pumps
		Biofilm formation
		Genomic structural variations
		Activation of stress response pathways
Parasites	Genome reduction	Extreme genomic compression
		Nucleolar proteome compression
		Genomic compactness and loss of metabolic pathways
	Surface antigen variation	Antigenic variation and recombination
		Amplification and loss of surface protein gene families
		Antigen switching and molecular mimicry
		MicroRNA and surface protein interactions
	Host-adaptive gene regulation	Epigenetic regulation
		Transcriptional regulation
		Signal transduction
		Post-translational regulation
Viruses	High mutation rate	Continuous adaptive evolution
		Genomic adaptive changes
		Non-synonymous mutations
	Gene recombination/rearrangement	Recombination among homologous viruses
		Recombination between different genotypes
		Cross-species recombination
	Vaccine escape	Antigenic drift and variation
		Genotype replacement
		Transmission and variation of vaccine virus strains
		Viral evolution under immune pressure
Prions	Protein misfolding	Enrichment of gut microbiota
		Dysbiosis of gut microbiome
	Host gene influence	Host gene susceptibility
		Host gene diversity
		Host gene polymorphism
	Cross-species transmission	PrP sequence origin
		PrP gene polymorphism

Hierarchical overview of the genomic evolutionary mechanisms that underlie pathogenicity in major intestinal microbes. The table is divided into three sections:

1. Primary microbial group—the five principal taxa examined.

2. Evolutionary subcategory—broad genomic processes characteristic of each group (e.g., horizontal gene transfer in bacteria, antifungal resistance in fungi).

3. Specific mechanisms—molecular and adaptive sub-processes such as conjugation, transduction, antigenic variation, and biofilm formation.

By bridging fundamental genomic research with clinical implementation, this review seeks to contribute to the advancement of precision diagnostics for enteric pathogens and enhance global public health preparedness.

## 2 Genomic evolutionary mechanisms of intestinal pathogens

### 2.1 Bacteria

Bacterial pathogens in the human gut play a critical role in gastrointestinal diseases by modulating host metabolism, immune responses, and digestive processes. Among these, *Enterobacteriaceae*—particularly *Escherichia coli* (*E. coli*) and *Klebsiella* spp.—are enriched in the gut microbiota of individuals with Crohn's disease and ulcerative colitis. Over the past decade, their genomes have demonstrated marked evolutionary plasticity, largely driven by virulence genes and mobile genetic elements (MGEs) that enhance pathogenicity across diverse host environments (Zhang J. et al., 2023). Key evolutionary mechanisms include horizontal gene transfer (HGT), recombination, regulatory network reprogramming, and antibiotic resistance development (Garud et al., 2019).

HGT facilitates rapid acquisition of virulence traits and reconfigures bacterial genomes by integrating genetic elements such as plasmids, bacteriophages, and transposons, thereby promoting adaptation to host-derived stresses. For example, *E. coli* O157:H7 acquired Shiga toxin genes (*stx*) via phage-mediated HGT, resulting in potent cytotoxicity and epithelial damage (Greig et al., 2020). Conjugative plasmids such as pVir mediate the transfer of master regulators like *hilD*, which activate type III secretion system 1 (TTSS-1), enhancing epithelial invasion and intracellular survival (Bakkeren et al., 2022). Bacteriophages, central to HGT, integrate into bacterial chromosomes as prophages and are induced by host stressors (e.g., oxidative bursts, antibiotics), entering lytic cycles that disseminate virulence factors between species (Frazao et al., 2019). Many prophage-encoded virulence genes exhibit phase variation, enabling dynamic expression in response to intestinal cues, thereby supporting immune evasion and persistent colonization.

Insertion sequence (IS) amplification—for example, IS1 and IS2 in *Shigella* spp.—drives genome reduction by disrupting or deleting functional genes, facilitating niche-specific adaptation and host specialization (Hawkey et al., 2020). These IS elements also cause chromosomal rearrangements affecting virulence gene expression. For instance, IS-mediated modifications in regulatory regions may upregulate or suppress invasion proteins, optimizing pathogenic potential. Such genome reduction enhances fitness in the intestinal niche by supporting immune evasion and colonization.

Beyond HGT, bacterial pathogens adapt by reprogramming metabolic and regulatory networks to resist environmental and host immune pressures. Adherent-invasive *E. coli* (AIEC), for example, acquires genomic islands encoding stress regulators such as *yfcV*, which confer oxidative stress resistance and promote survival within macrophage phagosomes. This tight linkage between virulence and persistence extends to regulation of biofilm formation and toxin production, reflecting multifactorial survival strategies in fluctuating gut environments.

Virulence evolution is also mediated by recombination and regulatory remodeling (Zhou et al., 2023). Unlike HGT, these processes modulate gene expression without sequence acquisition. Bacteria utilize promoter mutations, antisense RNAs, and epigenetic modifications to rapidly and reversibly regulate virulence genes. For instance, *Klebsiella* spp. control capsule polysaccharide synthesis via phase variation in *rmpA*, balancing immune evasion and genetic exchange to maintain adaptability (Wei et al., 2025). In *Salmonella*, the CRISPR-Cas system suppresses foreign DNA and concurrently represses *hilD*—a TTSS-1 regulator—linking immune defense to virulence attenuation (Sharma et al., 2024). Similarly, in *Vibrio parahaemolyticus*, the transcription factor QsvR synchronizes quorum sensing and virulence gene expression, coordinating responses to bile salts and mucus gradients to facilitate colonization (Zhang et al., 2021).

In enterohemorrhagic *E. coli* (EHEC) O157:H7, the two-component system UvrY functions as a central regulator, activating both the locus of enterocyte effacement (LEE) and non-LEE-encoded virulence genes, exemplifying a “master regulator–modular target” model (Wu et al., 2023). Under magnesium-limited conditions—commonly induced by host inflammation—sensor kinases phosphorylate UvrY, triggering LEE effector expression to promote epithelial adherence and secretion system activation. This signal-responsive network shifts resource allocation from growth to virulence, enhancing colonization under inflammatory stress.

Antimicrobial resistance (AMR) represents a critical global health threat extending beyond pediatric populations. The 2024 Global Burden of Disease study reported 1.26 million deaths directly attributable to AMR in 2021 and 4.95 million deaths associated overall (95% UI: 1.01–1.51 million and 3.95–5.70 million, respectively; Collaborators, 2024). Since 1990, AMR-related mortality has increased by over 100%, with disproportionate impacts in South Asia and Latin America. Projections estimate up to 1.91 million direct AMR deaths by 2050, particularly among the elderly, underscoring the urgency for preventive measures, vaccination, and novel antimicrobial development.

In gut pathogens, resistance typically follows a hierarchical trajectory: initial chromosomal mutations confer low-level resistance (e.g., *gyrA* mutations conferring fluoroquinolone resistance), followed by HGT-mediated acquisition of high-level resistance genes (e.g., *qnr* plasmids), and compensatory mutations that mitigate fitness costs. Although children in resource-limited regions are especially vulnerable, AMR constitutes a widespread and escalating global concern (Okumu et al., 2025).

Resistance emerges via intrinsic, acquired, and adaptive mechanisms (Elshobary et al., 2025). A notable example is the overexpression of resistance-nodulation-division (RND)-type efflux pumps, such as CmeABC in *Campylobacter* spp., which are upregulated by host-derived antimicrobial peptides and expel multiple antibiotics (Dai et al., 2024). While intrinsic resistance mechanisms represent baseline features, their amplification often results from regulatory mutations, contributing to multidrug resistance (MDR). Biofilm formation by *E. coli* also enhances resistance by limiting antibiotic penetration and reducing metabolic activity, yielding up to 1,000-fold increased tolerance, partly mediated by β-lactamase expression (Nasrollahian et al., 2024). Additional mechanisms include target site modification (e.g., *rpoB* mutations conferring rifampicin resistance), enzymatic degradation (e.g., extended-spectrum β-lactamases), and altered membrane permeability. HGT remains central to resistance dissemination, as exemplified by the co-localization of resistance genes on conjugative plasmids and integrons in *Shigella* spp. and non-typhoidal *Salmonella* (NTS) serovars (Wallace et al., 2020). These plasmids frequently co-transfer virulence and resistance genes—for example, *bla*_CTX − M−15_ alongside *iroN*—facilitating the emergence of hypervirulent, multidrug-resistant clones.

This co-evolution of resistance and virulence, driven by host and therapeutic pressures, enables pathogens to maintain colonization, invasion, and immune evasion despite antimicrobial exposure. Environmental stressors, such as climate change and pollution, further accelerate mutation rates and promote HGT via SOS response induction. Agricultural runoff containing heavy metals selects for metal resistance genes co-located with antibiotic resistance genes on MGEs, driving co-selection and sustaining resistance reservoirs even in the absence of antibiotic pressure.

In summary, gut bacterial pathogens evolve through HGT, regulatory remodeling, and resistance acquisition, resulting in extensive genomic plasticity that complicates clinical management. The convergence of virulence and resistance necessitates integrated surveillance systems monitoring both AMR determinants and virulence factors. A deeper understanding of these evolutionary mechanisms will facilitate the identification of genomic biomarkers (e.g., IS26 transposition sites) and inform the development of precision diagnostics. Novel therapeutic strategies—such as conjugation-blocking peptides, SOS response inhibitors, and CRISPR interference (CRISPRi) targeting key virulence regulators—represent promising approaches. Future research should prioritize longitudinal tracking of within-host pathogen evolution to inform personalized treatment regimens.

### 2.2 Fungi

Gut fungi predominantly adapt through genome remodeling and lineage-specific gene family diversification, in contrast to the horizontal gene transfer (HGT) mechanisms central to bacterial evolution. For instance, *Candida* adhesins evolve via recombination-driven diversification, demonstrating functional convergence across kingdoms under intense intestinal selective pressures. Elucidating these fungal adaptation strategies is essential for understanding infection dynamics and host–pathogen interactions.

Although less abundant than bacteria, gut fungi significantly influence host physiology and pathogenesis. Rather than relying on HGT, fungi adapt through structural genome variation, gene family expansion, and acquisition of antifungal resistance—mechanisms facilitating their transition from commensals to opportunistic pathogens within the complex intestinal ecosystem characterized by oxygen gradients, bile salts, and interkingdom competition (Huang et al., 2024).

Genomic structural variation constitutes a core mechanism underlying fungal adaptability and virulence regulation. Aneuploidy, chromosomal translocations, copy number variations (CNVs), and homologous recombination modulate both drug resistance and pathogenicity. These rearrangements are frequently induced by host-derived stressors; for example, bile acids induce DNA damage in *Candida*, while neutrophil-generated reactive oxygen species promote recombination in *Candida glabrata*.

In *Candida albicans*, chromosome 7 trisomy—commonly observed in the gut—elevates *NRG1* dosage, a transcriptional repressor of hyphal formation. This promotes a yeast-phase-locked phenotype, facilitating mucosal colonization and immune evasion (Kakade et al., 2023). Under azole stress, *C. albicans* acquires aneuploidies involving chromosomes 3, 4, or 5, which harbor resistance genes such as *ERG11, TAC1*, and *MRR1*. The resulting gene dosage effects upregulate efflux pumps and drug targets, conferring rapid fluconazole tolerance (Wang et al., 2025).

In *Cryptococcus* species, recombination hotspots within transposon-dense centromeres drive interchromosomal translocations. Amplification of the transcription factor *RYP2* enhances yeast morphology and elevates virulence through upregulation of adhesins and capsule biosynthesis (Voorhies et al., 2021). Environmental cues such as pH shifts and nutrient depletion further induce rearrangements in *C. albicans*, including tandem duplications of *SAP* protease genes and deletions of *FCR* family genes, thereby improving metabolic plasticity and adaptation to nutrient-limited niches (Todd et al., 2019).

Fungal virulence is further shaped by lineage-specific expansion and structural diversification of gene families, primarily mediated by endogenous mechanisms such as ectopic recombination, retrotransposition, and unequal crossing-over, particularly within subtelomeric regions characterized by elevated recombination activity.

In *C. albicans*, adhesin families such as *ALS* and *HYR/IFF* expand through recombination near chromosome ends, generating structural diversity within intrinsically disordered tandem repeats. This results in strain-specific adhesion profiles that optimize binding to mucins, epithelial surfaces, and abiotic substrates—enhancing biofilm formation, tissue tropism, and infection severity (Smoak et al., 2023). Similarly, *Candida parapsilosis* isolates from neonatal guts exhibit extensive CNV of *RTA3*, encoding a lipid translocase. Elevated *RTA3* copy number alters membrane rigidity, increasing resistance to azoles and cationic peptides, linking membrane remodeling to immune evasion (West et al., 2021). In clinical *C. albicans* strains, elevated *SAP* gene copy numbers correlate with increased tissue invasiveness in murine models, while allelic diversity within *SAP* genes further modifies substrate specificity and proteolytic activity, contributing to niche adaptation (Zoppo et al., 2021).

Antifungal resistance in gut fungi emerges via a hierarchical, multi-step process: initial point mutations reduce drug binding affinity, followed by gene amplification enhancing efflux pump and target enzyme expression, culminating in biofilm-mediated drug tolerance. The gastrointestinal tract serves as a reservoir for resistant fungal populations.

In *C. albicans*, missense mutations in *ERG11* (e.g., G464S) that reduce azole binding affinity often arise *de novo* during intestinal azole exposure. Biofilms further potentiate resistance through physical drug exclusion, overexpression of pumps such as *CDR1* and *MDR1*, and formation of metabolically quiescent persister cells exhibiting 100–1,000-fold increased drug tolerance (Lee et al., 2023). Expression of *CDR1* is regulated by the transcription factor *TAC1*, with both gain-of-function mutations and gene duplication at this locus contributing to high-level resistance (Kaur and Nobile, 2023; Ibe and Pohl, 2024).

Bacterial–fungal interactions also modulate antifungal resistance. For example, *Enterococcus* spp. secrete farnesol, which inhibits *Candida* biofilm dispersal, while *Pseudomonas aeruginosa* releases pyocyanin that induces *CDR1*, enhancing azole resistance. These interkingdom dynamics complicate therapeutic management in polymicrobial gut environments.

Additional resistance mechanisms include upregulation of alternative efflux pumps (*MDR1, FLU1*), alterations in membrane sterol synthesis via mutations in *ERG3* and *ERG6*, and activation of stress response pathways that enhance fungal survival under oxidative and membrane stress. Collectively, these adaptations facilitate fungal persistence despite antifungal treatment.

In summary, gut fungi exhibit remarkable adaptive potential through chromosomal remodeling, gene family diversification, and antifungal resistance evolution. These mechanisms fundamentally differ from bacterial strategies based on horizontal gene acquisition. Fungal aneuploidy involves metabolic trade-offs balancing growth and virulence, while adhesin variation enables niche-specific colonization. Together, these strategies shape the evolutionary trajectory of fungal pathogens within the gut. Targeting fungal genome instability—such as suppressing aneuploidy or recombination hotspots—represents a promising therapeutic approach. Future research integrating multi-omics and *in vivo* models is essential for elucidating convergent evolutionary mechanisms shaped by host–microbe interactions.

### 2.3 Parasites

Intestinal parasites, particularly protozoans, have evolved distinct adaptive trajectories under the selective pressure of obligate parasitism. Their pathogenicity is primarily driven by three core evolutionary strategies: extreme genome reduction, antigenic variation, and modulation of host regulatory networks. These mechanisms, arising from prolonged host–parasite coevolution, collectively underpin persistent colonization and immune evasion.

Genome compaction represents a hallmark of parasitic adaptation, characterized by the elimination of redundant metabolic pathways and the conservation or amplification of genes essential for virulence. *Encephalitozoon cuniculi*, an obligate intracellular microsporidian, harbors a highly reduced genome (< 3 Mbp)—substantially smaller than those of many prokaryotes—with extensive loss of central metabolic processes, including the tricarboxylic acid (TCA) cycle and mitochondrial respiration (Zarsky et al., 2023). Devoid of autonomous ATP synthesis, this parasite relies entirely on host-derived energy, facilitated by conserved invasion machinery such as polar tube proteins (PTP1–5) and hexokinases. This metabolic parasitism enhances replication efficiency and exacerbates virulence, particularly in immunocompromised hosts.

*Giardia lamblia* exhibits analogous genomic streamlining. Its nucleolar proteome contains only ~147 proteins, predominantly dedicated to ribosome biogenesis (e.g., fibrillarin, nucleolin), thereby supporting efficient protein translation while eliminating non-essential nuclear functions (Feng et al., 2020). This compact proteomic organization enables rapid trophozoite proliferation during intestinal colonization.

*Cryptosporidium* spp. have undergone further extensive genome reduction, completely abolishing mitochondrial oxidative phosphorylation. Their mitosomes retain only iron–sulfur (Fe–S) cluster assembly functions, enforcing strict reliance on host-derived ATP (Arias-Agudelo et al., 2020). Their streamlined genomes prioritize the expression of effector molecules critical for invasion and survival, including mucin-like GP900 for epithelial adhesion, thrombospondin-related adhesive protein TRAP-C1 for gliding motility, and dense granule proteins (GRAs) that restructure host epithelial cells to form parasitophorous vacuoles, thereby facilitating intracellular persistence and immune evasion.

To evade host adaptive immunity, protozoan parasites employ antigenic variation systems that dynamically modulate their surface antigen profiles. *Entamoeba histolytica* expresses multiple isoforms of Gal/GalNAc lectins—surface adhesins mediating mucin binding, epithelial cell attachment, and cytotoxicity. Isoform switching alters antigenic presentation, directly facilitating immune evasion. Additionally, *E. histolytica* secretes cysteine proteases that degrade secretory immunoglobulin A (IgA) and extracellular matrix components, impairing mucosal defense mechanisms and promoting tissue invasion (Marie and Petri, 2014).

*Blastocystis* spp., particularly subtype ST6, also secrete cysteine proteases that degrade IgA and modulate host immune signaling. These enzymes induce interleukin-8 (IL-8) expression, recruiting polymorphonuclear leukocytes (PMNs) and initiating localized inflammation. Concurrently, elevated levels of T helper 1 (Th1) cytokines—including interleukin-12 (IL-12) and interferon-gamma (IFN-γ)—reflect a cell-mediated immune response that facilitates chronic colonization. Subtypes ST2 and ST6 are notably correlated with increased proteolytic activity, upregulated interleukin-6 (IL-6) secretion, and symptomatic infection. Such inflammatory responses may predispose hosts to irritable bowel syndrome (IBS), linking *Blastocystis*-induced immune modulation to chronic gastrointestinal pathology (Karamati et al., 2021).

Beyond immune evasion, intestinal parasites manipulate host immune function through the secretion of immunomodulatory molecules. *Trichuris trichiura* (whipworm) releases excretory–secretory (ES) products—including the p43 protein, short-chain fatty acids (e.g., acetate, butyrate), and complex glycans—that interact with Toll-like receptor (TLR)-expressing dendritic cells. These signals suppress tumor necrosis factor-alpha (TNF-α) production, inhibit dendritic cell maturation, and attenuate Th1/Th17 responses, while promoting interleukin-10 (IL-10) secretion and mucosal tolerance. Although regulatory T cell (Treg) expansion is modest compared to other helminths, the induced anti-inflammatory milieu supports sustained colonization. Notably, the immunomodulatory activity of short-chain fatty acids may be enhanced by the parasite-associated microbiota (Shears and Grencis, 2022).

Structural adaptations further enhance persistence. *Giardia lamblia* utilizes a ventral adhesive disc composed of microtubule–actin complexes to maintain tight adhesion to intestinal epithelium and resist peristaltic clearance. The actin-like protein GlActin is essential for disc structural integrity and function; its depletion disrupts attachment. Disc and actin-associated protein 1 (DAAP1), localized to the ventral groove, regulates fluid dynamics to stabilize adhesion under shear stress. While DAAP1 is not required for disc morphogenesis, its absence significantly impairs attachment strength and colonization efficiency in murine models (Steele-Ogus et al., 2022).

In summary, parasitic protozoa rely on genomic minimality, antigenic plasticity, and modulation of host regulatory networks to establish chronic infections within the gastrointestinal tract. Unlike bacteria, which frequently acquire virulence traits via horizontal gene transfer, or fungi, which depend on chromosomal rearrangements, parasites exemplify an alternative evolutionary strategy—achieving functional complexity through reductive genomic evolution. From energy harvesting in *Cryptosporidium* to proteomic efficiency in *Giardia lamblia*, these adaptations illustrate how parasitic lifestyles are optimized via streamlined genetic architectures. Elucidation of these mechanisms provides critical insights into conserved evolutionary strategies across biological kingdoms and identifies promising targets for therapeutic intervention.

### 2.4 Viruses

Within the intestinal ecosystem, viruses constitute highly adaptable pathogenic agents, with their pathogenicity primarily governed by three core genomic strategies: hypermutation, genetic recombination, and structural optimization for immune evasion. Collectively, these evolutionary mechanisms mediate immune escape, expand tissue tropism and host range, and augment viral persistence and transmissibility across diverse host environments.

RNA viruses such as *norovirus* GII.4 exemplify mutation-driven adaptation. Owing to the absence of proofreading activity in RNA-dependent RNA polymerases (RdRps), *noroviruses* exhibit mutation rates ranging from 10^−3^ to 10^−5^ substitutions per nucleotide per replication cycle, generating extensive quasispecies swarms that enable rapid phenotypic adaptation. In *norovirus* GII.4, mutations predominantly accumulate in the VP1 major capsid protein, particularly within antigenic epitopes (Sites A–G), inducing subtle structural alterations that evade neutralizing antibodies while preserving affinity for histo-blood group antigens (HBGAs) expressed on intestinal epithelial cells. Concurrent mutations in the VP2 (p22) minor capsid protein enhance virion stability and replication fidelity, conferring additional fitness advantages under immune pressure (Kistler and Bedford, 2023).

Similarly, *rotavirus* A (e.g., strain G9P[8]) undergoes immune-driven antigenic evolution via amino acid substitutions in the VP7 glycoprotein, specifically within antigenic regions (Sites 7–8), which reduce antibody-mediated neutralization. Segmental reassortment between VP4 and VP7 genes further diversifies antigenic profiles, generating variants capable of reinfecting previously vaccinated individuals. *Astrovirus* MLB2 adapts through remodeling of the capsid P2 domain, which enhances HBGA binding affinity and alters viral uncoating kinetics, thereby increasing intestinal tropism and fecal shedding. These capsid adaptations balance immune evasion with infectivity, supporting persistence in hosts with pre-existing immunity (Liu X. et al., 2022).

Zoonotic enteric viruses exhibit heightened mutational plasticity. For instance, rodent-borne *rosavirus* demonstrates a VP1 mutation rate of 2.12 × 10^−3^ substitutions per site per year, with mutations concentrated in hydrophobic receptor-binding regions. Deep sequencing analyses reveal variant-rich quasispecies swarms with expanded receptor usage, indicating an augmented potential for cross-species transmission (Zhang et al., 2024).

Beyond point mutations, genetic recombination drives abrupt evolutionary shifts. *Rotavirus* A frequently undergoes segmental reassortment and homologous recombination, particularly within the VP1 polymerase gene, where recombination breakpoints cluster in conserved RNA stem-loop regions (nucleotides 800–850), facilitating template switching. Recombinant G9P[8]-DS-1-like strains exhibit enhanced capsid stability (ΔG = −3.2 kcal/mol) and escape from dominant neutralizing epitopes, promoting persistence despite widespread population immunity (Hoxie and Dennehy, 2020).

In *noroviruses*, recombination events near the ORF1/ORF2 junction generate hybrid capsids that integrate non-structural and structural proteins. These chimeric P2 domains expand HBGA-binding capabilities and enhance mucosal adherence, increasing fecal shedding and transmission efficiency—particularly in pediatric populations and high-density settings (Ludwig-Begall et al., 2021; Cannon et al., 2021).

Cross-species recombination events pose significant zoonotic risks. The human–porcine reassortant *rotavirus* strain HB05 (G9P[23]) incorporates a human-derived VP7 segment with a porcine-origin VP4, conferring dual-species receptor tropism. This strain replicates 3.5-fold more efficiently in human intestinal organoids and induces 89% mortality in neonatal piglets, underscoring its heightened virulence potential (Li et al., 2025). Similarly, neurotropic *astrovirus* recombinants (e.g., MLB1–VA1) harbor capsid modifications that enhance sialic acid binding affinity and facilitate neuronal cell entry, contributing to encephalitis in immunocompromised hosts (Roach and Langlois, 2021).

Vaccination imposes strong selective pressure that can inadvertently accelerate viral evolution. In *norovirus* GII.4, mutations within the VP1 P2 domain (e.g., A358N, D9YIN, S59MD, T97A) alter epitope conformation, surface hydrophobicity, and electrostatic charge, preserving HBGA-binding affinity while reducing antibody recognition. Notably, Site A (residues 294–298) is critical for immune evasion and vaccine escape, contributing to breakthrough infections even in vaccinated cohorts (Carlson et al., 2024).

Live-attenuated *rotavirus* vaccines (RVVs) impose additional evolutionary constraints. Post-vaccination viral shedding facilitates reassortment between vaccine-derived and wild-type strains, while immune pressure selects for emerging genotypes. Novel variants such as G12P[6] exhibit modified VP4/VP7 antigens and enhanced immune evasion properties, contributing to increased breakthrough infection rates (Mhango et al., 2023).

Recombinant *astrovirus* strains carrying mink-derived capsid segments demonstrate expanded organotropism, leveraging upregulated platelet-derived growth factor receptor alpha (PDGFRα) to infect human neural progenitor cells, resulting in extra-intestinal manifestations such as encephalitis (El-Heneidy et al., 2023).

*Porcine epidemic diarrhea virus* (PEDV) illustrates vaccine-induced lineage replacement. G2 strains have supplanted G1 variants via mutations in the S1 domain that increase sialic acid affinity and alter COE neutralizing epitopes, thereby evading G1-derived maternal antibodies and causing >95% mortality in piglets—highlighting how vaccine-driven immune selection can enhance viral pathogenicity (Zhang H. et al., 2023).

Collectively, immune pressure channels viral evolution along adaptive landscapes, where antigenic changes may outpace host immunological memory. The emergence of novel *norovirus* GII.4 variants is frequently marked by amino acid insertions and shifts in charge/hydrophobicity within the VP1 P2 domain, preserving HBGA binding while reducing susceptibility to neutralization. Although quantitative estimates of >40% vaccine efficacy loss remain limited, these antigenic dynamics present substantial challenges for effective vaccine design (Chhabra et al., 2024).

In summary, the genomic evolution of enteric viruses—driven by mutation, recombination, and immune selection—enhances their virulence, transmissibility, and zoonotic potential. Addressing these challenges requires a multifaceted strategy encompassing: (1) global genomic surveillance of emerging variants; (2) rational vaccine design targeting conserved, functionally constrained epitopes (e.g., *norovirus* HBGA-binding sites, *rotavirus* VP6); (3) antiviral strategies exploiting error-prone replication (e.g., lethal mutagenesis); and (4) ecological interventions to reduce animal–human transmission interfaces. Recognizing enteric viruses as dynamic quasispecies is critical for guiding public health policy and strengthening pandemic preparedness.

### 2.5 Prions

Prions are unique infectious agents comprising solely misfolded proteins, devoid of nucleic acid components. Their pathogenicity originates from the conformational conversion of host-encoded cellular prion protein (PrP^∧^C) into a pathogenic, self-propagating isoform (PrP^∧^Sc), which assembles into highly stable, transmissible fibrils. Although prion diseases—such as bovine spongiform encephalopathy (BSE) and Creutzfeldt–Jakob disease (CJD)—are classically defined by fatal central nervous system (CNS) neurodegeneration, the gastrointestinal (GI) tract serves as a critical reservoir for prion entry, persistence, and neuroinvasion. Following oral exposure, prions establish long-term persistence within gut-associated lymphoid tissues (GALT), most notably in Peyer's patches, where they leverage mucosal immune tolerance mechanisms to evade clearance. Subsequent retrograde transport via the enteric nervous system (ENS) facilitates neuroinvasion, underscoring the GI tract as a pivotal determinant of prion pathogenesis. The evolutionary trajectory of prion pathogenicity is governed by three interrelated processes: (i) microenvironmental modulation of misfolding kinetics, (ii) host genetic determinants of susceptibility and phenotypic expression, and (iii) polymorphic constraints on cross-species transmissibility.

The intestinal lumen represents a complex, dynamic microenvironment in which the gut microbiota and their metabolites profoundly modulate prion misfolding dynamics and disease progression. Microbial dysbiosis has been identified as a critical modulator of prion pathogenesis through multiple convergent pathways: disruption of epithelial barrier integrity, reduction of protective short-chain fatty acids (SCFAs; e.g., butyrate), elevation of pro-inflammatory molecules such as lipopolysaccharide (LPS), and cross-seeding of prion aggregation by bacterial amyloid proteins (e.g., Curli; Mahbub et al., 2024). These alterations promote PrP^∧^Sc formation and stabilization within the intestinal milieu. Notably, while certain SCFA-producing bacteria (e.g., *Lachnospiraceae, Ruminococcaceae*) are typically regarded as beneficial, their expansion may paradoxically exacerbate neuroinflammation via microglial activation, indirectly supporting PrP^∧^Sc persistence. Conversely, increased abundance of SCFA-suppressing genera such as *Bilophila* has been associated with impaired hippocampal synaptic plasticity, potentially accelerating prion dissemination (Losa et al., 2024). Additionally, accumulation of neurotoxic microbial metabolites—including D-lactate and ammonia—further disrupts the gut–brain barrier and promotes CNS inflammation, thereby facilitating PrP^∧^Sc propagation along the gut–brain axis (Islam et al., 2024). Collectively, these findings highlight the central role of the intestinal microbiota in regulating prion conformational transitions and neuropathological outcomes.

At the host genomic level, polymorphisms in the PRNP gene constitute primary determinants of prion disease susceptibility, incubation period, and clinicopathological manifestations. Over 50 pathogenic PRNP mutations have been reported worldwide, underlying familial prion disease variants. For example, the E200K mutation is frequently associated with familial CJD in European populations; the D178N mutation, in conjunction with codon 129 polymorphism, dictates phenotypic divergence between genetic CJD and fatal familial insomnia; the P102L mutation is linked to Gerstmann–Sträussler–Scheinker syndrome with variable expressivity; and the rare R208H mutation has been described in cases resembling progressive supranuclear palsy. Octapeptide repeat insertions (OPRIs) exhibit variable phenotypic outcomes contingent upon both repeat number and codon 129 status. Homozygosity at codon 129 (MM or VV) significantly increases susceptibility to both acquired and inherited prion diseases, with nearly complete penetrance observed in many familial cases (Jankovska et al., 2021). In sporadic CJD (sCJD), codon 129 genotype acts as a key modifier: MM homozygosity predominates among patients (~60% in Barcelona, ~71% in Bologna), whereas heterozygosity (MV) confers significant protective effects, presumably by reducing the efficiency of PrP^∧^C-to-PrP^∧^Sc conformational conversion (Gelpi et al., 2022). In cervids with chronic wasting disease (CWD), PRNP polymorphisms (e.g., S96, H95) modulate susceptibility, incubation period, prion burden, and strain properties. CWD strains further demonstrate adaptive evolution in novel hosts (e.g., the H95′ strain in tg60 transgenic mice), emphasizing the critical role of PRNP diversity in intra- and interspecies prion adaptation (Duque Velasquez et al., 2020).

Cross-species transmission represents a major evolutionary mechanism in prion biology, primarily governed by structural compatibility between exogenous PrP^∧^Sc and host PrP^∧^C. This compatibility is determined by sequence homology and conformational dynamics influenced by host PRNP polymorphisms. Protein misfolding cyclic amplification (PMCA) assays evaluating the zoonotic potential of CWD prions reveal strict host-specific barriers. For instance, CWD prions from elk with 132MM or 132ML genotypes efficiently convert human 129VV PrP^∧^C but display minimal conversion of 129MM PrP^∧^C; elk 132LL prions demonstrate negligible conversion across genotypes. White-tailed deer (WTD)–derived prions exhibit even lower conversion efficiency for human 129VV PrP^∧^C substrates (Wang et al., 2021). Specific PRNP alleles modulate transmission potential: WTD genotypes 96SS and 95HH confer reduced susceptibility; the 225F allele in mule deer prolongs the incubation period; sheep haplotype VRQ facilitates scrapie transmission, while ARR confers protection. In humans, homozygosity for methionine at codon 129 markedly increases susceptibility to variant CJD. These polymorphisms reconfigure the conformational landscape of PrP^∧^C, influencing templating efficiency and conversion kinetics. Alleles such as 96S in WTD and 225F in mule deer function as molecular bottlenecks, emulating interspecies transmission barriers. Such polymorphic dynamics underpin the reported ability of CWD to infect diverse species—including *Sus scrofa* (swine), *Ovis aries* (sheep), and *Bos taurus* (cattle)—highlighting the pressing need for comprehensive risk assessment of its zoonotic potential (Moazami-Goudarzi et al., 2021).

In summary, prion evolution fundamentally differs from that of nucleic acid-based pathogens, being mediated solely through protein conformational transitions. This process is elaborately regulated by host genetic factors (notably PRNP polymorphisms) and the intestinal microenvironment. The GI tract not only acts as a critical site for prion uptake and initial propagation but also functions as a dynamic junction where microbial ecology and host genetics converge to determine misfolding efficiency, neuroinvasion capacity, and disease progression. Furthermore, polymorphic variations in host PrP^∧^C impose substantial—albeit frequently surmountable—barriers to cross-species transmission, enabling the adaptive evolution of novel prion strains with expanded host ranges and altered pathogenic properties. Elucidating the interactions between protein misfolding dynamics, host genetic predisposition, and interspecies transmission potential is critical for forecasting outbreak risks, identifying therapeutic targets (e.g., interventions against gut-phase amplification or PrP^∧^Sc formation), and reducing zoonotic risks posed by animal prion diseases such as CWD.

## 3 Virulence factor-based analysis Paradigm: *Escherichia coli* and beyond

### 3.1 *E. coli* as a model for virulence factor evolution and detection strategy

*Escherichia coli* stands as the preeminent Gram-negative model for virulence evolution research, leveraging its comprehensively annotated genome, unparalleled genetic tractability, and robust experimental versatility. Systematic dissection of its nine intestinal pathotypes—enteropathogenic *E. coli* (EPEC), enterohemorrhagic *E. coli* (EHEC), enterotoxigenic *E. coli* (ETEC), enteroinvasive *E. coli* (EIEC), enteroaggregative *E. coli* (EAEC), diffusely adherent *E. coli* (DAEC), adherent-invasive *E. coli* (AIEC), alongside emerging hybrid pathovars—reveals how horizontally transmitted mobile genetic elements orchestrate virulence arsenals. Plasmids, bacteriophages, and composite transposons encode EHEC's Shiga toxins (*stx1/stx2*), EPEC's type III secretion system effectors (EspA/B/D), and ETEC's colonizing factors (CFA/I/II/IV) coupled with heat-labile and heat-stable enterotoxins. These molecular adaptations exemplify real-time pathoadaptation driven by genomic plasticity, providing an evolutionary roadmap for enteropathogens (Pakbin et al., 2021).

Critically, *E. coli* exhibits bimodal genomic architecture characterized by ~1,000–3,000 essential core genes vs. dynamic accessory genomes enabling ecological diversification. Its eight phylogenomic groups (A, B1, B2, C, D, E, F, cryptic clade I) harbor hybrid pathogens that maintain equilibrium between virulence and commensalism. When integrated with single-cell omics and CRISPR-interference (CRISPRi) technologies, this model illuminates landscape-scale gene flux across microbial guilds, spatiotemporal dynamics of host-pathogen dialogues, and rational design of next-generation vaccines exemplified by MecVax against ETEC (Geurtsen et al., 2022). This cross-kingdom relevance extends to viral, prion, and bacterial pathogenesis studies, directly catalyzing diagnostic innovation through comparative pathoadaptation mechanisms.

### 3.2 Overview of VFDB and virulence factor selection

The Virulence Factor Database (VFDB) represents the definitive resource for virulence annotation (Liu B. et al., 2022). The 2022 release introduces transformative features including ontological restructuring into 14 universal categories such as adhesion, invasion, and toxin production, with over 100 subcategories resolving longstanding taxonomic biases. Expanded curation encompasses 1,885 adhesins, 391 invasins, and a novel immunoevasion class addressing functional redundancy. Computational optimization via a JavaScript-free interface accelerates large-scale queries, while standardized datasets enable machine learning-driven virulence prediction through homology searches and functional forecasting.

Our analysis leveraged 335 curated *Escherichia coli* virulence factors from VFDB spanning enteropathogenic *E. coli* (EPEC), Shiga toxin-producing *E. coli* (STEC), and emerging pathotypes. These molecular determinants encapsulate mobile genetic element-mediated evolutionary mechanisms detailed in Section 3.1, with phylogenomic interrogation revealing tempo-spatial patterns of virulence module dissemination across ecological gradients through recombination hotspots and selection signatures.

### 3.3 Functional annotation and molecular mechanism inference of *E. coli* virulence factors

To systematically characterize the functional modules and pathogenic mechanisms of *Escherichia coli* (*E. coli*) virulence factors, this study conducted high-throughput annotation and mechanistic inference of 335 representative virulence genes curated from the Virulence Factor Database (VFDB). We integrated three widely utilized bioinformatics platforms—COG (Clusters of Orthologous Groups), GO (Gene Ontology), and InterProScan—to link large-scale data mining with detailed molecular interpretation.

**Figure 1:**
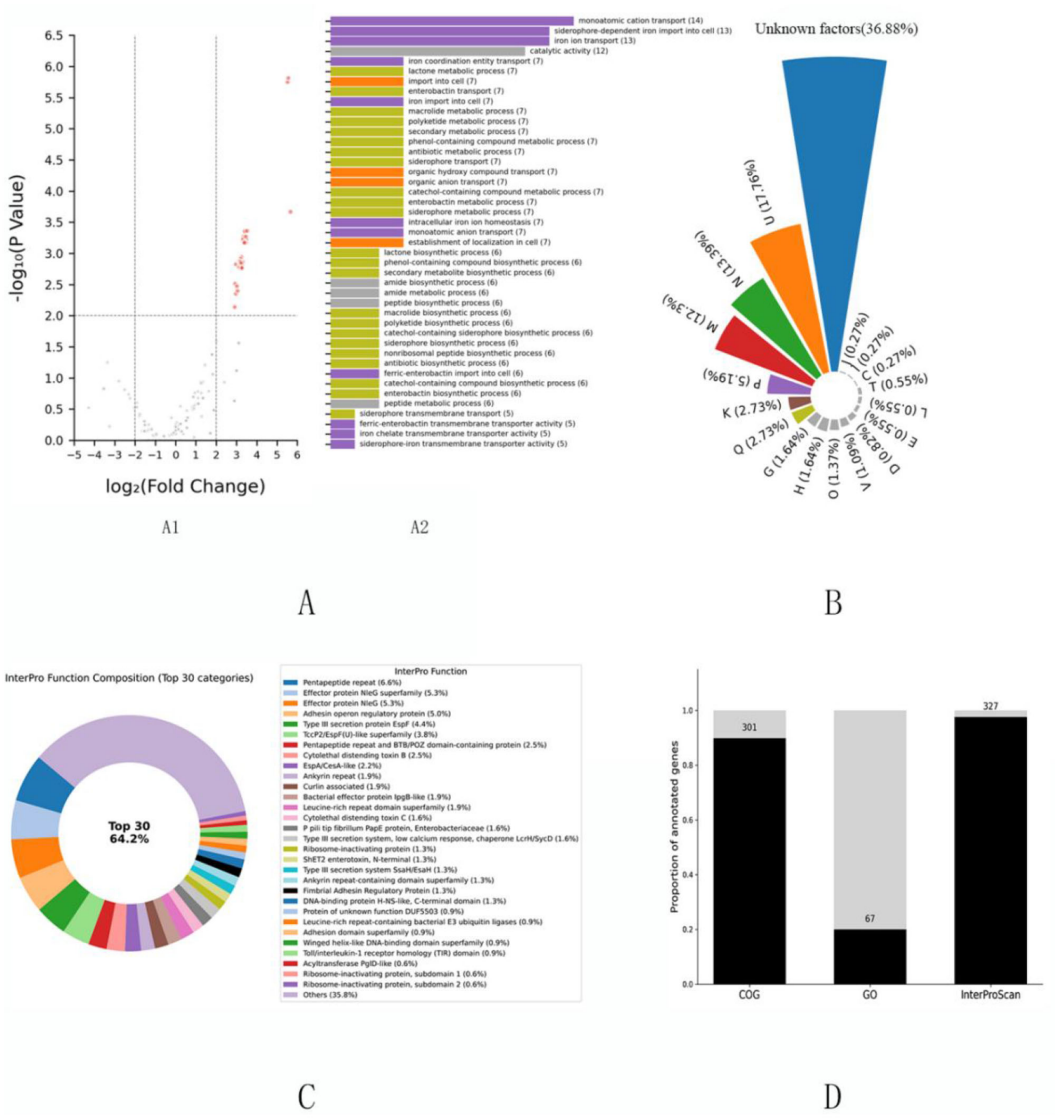
Functional annotation analysis of high-quality *Escherichia coli* virulence factors (*n* = 335). Integrative multi-dimensional functional annotation of 335 rigorously curated *Escherichia coli* virulence factors from the Virulence Factor Database (VFDB), presented in four panels **(A–D)**: **(A)** Differential functional enrichment analysis. (A1) Gene Ontology (GO) enrichment comparing high-confidence virulence factors to the full VFDB dataset, identifying 43 significantly upregulated genes (*P* < 0.01; detailed statistical methods in Methods). (A2) Functional categorization of these 43 genes shown as a bar chart, with colors corresponding to Clusters of Orthologous Groups (COG) categories in panel **(B)** to facilitate integrative cross-platform comparison. **(B)** COG-based functional annotation of all 335 virulence factors depicted as a pie chart showing proportional distribution across categories; color scheme matches panel (A2) for consistency. **(C)** InterProScan domain-based annotation of 135 virulence factors initially classified as “function unknown” by COG, with 125 successfully annotated. The 30 most frequent functional categories, ranked by occurrence, are visualized in a donut chart, highlighting core functional modules derived from protein domain analysis. **(D)** Comparative annotation coverage across COG, GO, and InterProScan platforms shown as a stacked bar chart, quantifying relative completeness and illustrating the complementary strengths of the combined approaches, emphasizing enhanced functional resolution via integration.

Initial annotation via the COG database was performed for 301 virulence factors, with GO functional terms assigned to 67 genes (Figure 1A); collectively, these platforms annotated 225 genes. An additional 110 genes were annotated by only one system, while 135 remained unannotated, highlighting significant functional ambiguity. The updated COG platform enhances annotation accuracy and cross-species consistency through RefSeq identifier stabilization, incorporation of over 200 new protein families, and reintroduction of detailed metabolic pathway classifications—particularly improving annotation of key virulence genes such as Shiga toxins (Figure 1B) (Galperin et al., 2021). Integration with InterProScan domain analysis assigned clear functional roles to 125 of the previously unannotated 135 factors, underscoring the utility of domain-based strategies in completing annotations. By integrating multiple signature databases and AlphaFold-predicted structures, InterProScan provides multilayered annotations (Figure 1C)—including GO terms for molecular function, biological process, and cellular component—thereby supporting robust virulence genomics and anti-infective discovery (Blum et al., 2025). In total, InterProScan successfully annotated 327 of the 335 virulence factors, significantly improving annotation coverage and reliability (Figure 1D).

#### 3.3.1 Effector and toxin-associated mechanisms

Approximately 21% of annotated factors are linked to classical effector or toxin functions, including families such as NleG, Shiga toxins, and cytolethal distending toxins (CDT). These factors disrupt host metabolism, cell cycle regulation, or immune responses. GO enrichment for “toxin metabolic process” and classification within COG category Q (secondary metabolite biosynthesis) further support their central role in virulence mechanisms (Marcos-Vilchis et al., 2025).

#### 3.3.2 Repeat domains and regulatory mechanisms

Roughly 18.1% of factors contain repeat motifs (e.g., ankyrin repeats, leucine-rich repeats), suggesting involvement in protein–protein interactions and DNA-binding activities related to virulence regulation. InterProScan identified H-NS-like DNA-binding regulators within this group, while COG category U classification implicates these proteins in intracellular transport and signal transduction.

#### 3.3.3 Adhesion and structural components

Approximately 9.4% of factors, including fimbrial adhesins (e.g., PapE), are implicated in bacterial adherence and structural assembly—processes essential for host colonization. GO enrichment analysis confirmed significant involvement of adhesion-related pathways.

#### 3.3.4 Secretory and nutrient acquisition systems

Around 10.1% of genes contribute to secretion systems (e.g., type III secretion system [T3SS]) and siderophore-mediated iron uptake (e.g., enterobactin receptors), with enrichment in COG categories P (secretion systems) and U. T3SS components (e.g., SsaH, SctQ) facilitate direct effector translocation into host cells to modulate immune responses, while siderophore systems confer nutritional advantages within host environments, positioning them as attractive anti-infective targets (Cavas and Kirkiz, 2022).

Additionally, 7.2% of genes were grouped into fragmented or poorly defined functional categories, including those encoding ribosome-inactivating domains, suggesting uncharacterized virulence mechanisms. Approximately 10 virulence factors remained unannotated by all methods, warranting further investigation via structural modeling and multi-omics approaches.

The integrated use of COG, GO, and InterProScan has substantially enhanced both the depth and resolution of *E. coli* virulence gene annotation. This comprehensive framework clarifies principal functional classes of virulence factors and establishes a scalable methodological basis for functional inference across diverse pathogenic taxa.

### 3.4 Extension to other intestinal Pathogens: resources and limitations

Following the multi-dimensional functional annotation and mechanistic classification of *Escherichia coli* (*E. coli*) virulence factors, this study evaluates the feasibility of extending this analytical paradigm to other major intestinal pathogens, including viruses, fungi, parasites, and prions. Despite divergent evolutionary trajectories and infection mechanisms, these pathogens universally rely on functionally defined virulence factors and pathogenic strategies for critical processes such as host invasion, immune evasion, and infection establishment. Cross-species conservation assessments of bacterial virulence factors—such as quorum-sensing systems (QSSs)—may yield critical insights for anti-virulence strategies targeting viruses or parasites, enabling the identification of shared mechanisms and facilitating the development of broad-spectrum anti-virulence agents. Additionally, methodologies including genome island detection, pathogen genome-wide association studies (GWAS), and associated functional validation pipelines may be adapted for non-bacterial pathogen research (Lau et al., 2023).

#### 3.4.1 Cross-species applicability of mainstream analytic platforms

Many mainstream analytic platforms are inherently designed for cross-species utility. For example, InterProScan performs protein function annotation based on structural domains and supports bacterial, eukaryotic, and viral proteins, making it suitable for identifying fungal virulence factors, viral structural proteins, and protozoan effectors. The Gene Ontology (GO) system, a taxonomically neutral functional classification standard, is widely used for annotating viral envelope proteins, fungal adhesins, and parasitic secretory factors. Metabolic pathway databases such as KEGG and Reactome further aid in identifying shared biological processes associated with virulence expression across pathogen types. KEGG, for instance, provides a genome browser integrating viral ortholog clusters (VOCs) and KEGG Orthology (KO) entries to support conserved order analysis and functional prediction of viral genes; recent “provisional KO” definitions extend its coverage in viral annotation (Homma et al., 2023).

With the increasing availability of high-precision protein structure prediction tools like AlphaFold, domain-based modeling has emerged as a powerful approach to compensate for genome annotation gaps. AlphaFold-Multimer (AFM) enables complex structure prediction, offering a framework to dissect cross-species interactions among virulence factors—a critical advantage where conventional homology-based methods fail. These structural insights provide a molecular basis for understanding host–pathogen interactions and disease resistance mechanisms in both humans and crops. Integrating structural genomics with evolutionary algorithms may further accelerate the co-development of interactomics and function prediction pipelines (Jin et al., 2023).

#### 3.4.2 Pathogen-specific barriers to implementation

Despite the potential for cross-application, pathogen-specific biological differences impose practical challenges. Viral genomes are typically short with frequent overlapping genes, complicating open reading frame (ORF) detection and function prediction. Viral proteins often lack homologous sequences, rendering traditional annotation tools ineffective. Fungi exhibit complex genome architectures, alternative splicing, and epigenetic regulation, demanding higher annotation accuracy. Protozoan parasites (e.g., *Cryptosporidium, Plasmodium*) display pronounced stage-specific gene expression, with many virulence factors restricted to specific life stages, challenging static annotation methods. Prions, as non-nucleic acid infectious agents, lie entirely outside sequence-based analysis, requiring structure modeling and biophysical experiments. While platforms like Companion address issues such as inaccurate gene models, limited RNA sequencing (RNA-Seq) support, or poor-quality reference genomes through genome alignment and visualization tools, challenges persist due to the lack of standardized evaluation criteria, high interspecies sequence variability, and strict International Nucleotide Sequence Database Collaboration (INSDC) submission requirements (Haese-Hill et al., 2024).

Several pathogen-specific databases support functional annotation across diverse pathogen types:

(1) **PHI-base (Pathogen–Host Interactions Database):** Launched in 2005, PHI-base is an open-access database cataloging experimentally verified pathogen–host interaction phenotypes, including virulence genes and effector proteins in fungal, bacterial, and protozoan pathogens. Adhering to FAIR principles (Findable, Accessible, Interoperable, Reusable), it supports cross-species comparative analysis and facilitates target discovery in medicine, agriculture, and ecology (Urban et al., 2022).
(2) **EuPathDB:** Integrating genomic, transcriptomic, and phenotypic data for 22 eukaryotic parasites (e.g., *Leishmania, Toxoplasma, Trypanosoma*), EuPathDB enables virulence factor screening via multi-step ortholog-based search strategies. For example, *Plasmodium*-derived genes can guide the identification of apicoplast-targeting genes in *Toxoplasma*. The platform offers over 80 search functionalities and user annotation tools, supporting biosecurity and global health initiatives. Future updates will incorporate phenotype and metabolome datasets to enhance discovery capabilities (Aurrecoechea et al., 2010).
(3) **ViPR (Virus Pathogen Database and Analysis Resource):** Supported by the U.S. National Institute of Allergy and Infectious Diseases (NIAID) Bioinformatics Resource Center (BRC), ViPR integrates multi-viral family data, including sequence records, gene/protein annotations, 3D structures, immune epitopes, clinical metadata, and comparative genomics results. It serves as a comprehensive resource for virology research, supporting diagnostics, therapeutics, and preventive strategies for high-priority and emerging viral pathogens.
(4) **PrionHome:** Compiling ~2,000 sequences, PrionHome uses N/Q-bias analysis and hidden Markov model (HMM) algorithms to identify prion-related proteins, including known prionogenic sequences (PrP, Sup35p, Ure2p), candidate prion sequences, and their homologs. It analyzes key features (N/Q-rich domains, YYR motifs, intrinsically disordered regions, structural elements) to aid prion candidate screening. Case studies demonstrate that yeast prion sequences exhibit strong N/Q enrichment and retain characteristic motifs (e.g., YYR in PrP), while interaction partners (e.g., GPR1) can induce prion formation via cross-seeding. Users can efficiently search via BLAST or SQL queries; future updates plan to incorporate polymorphism data for enhanced functionality (Harbi et al., 2012).

Despite inherent biological differences among pathogen classes, the annotation and mechanistic classification framework developed for *E. coli* virulence factors offers significant translational value, reflected in three key aspects:

(1) Broad Applicability of Domain-Based and Phylogenetic Tools: Platforms like InterProScan, GO, and COG leverage structural domains and evolutionary conservation, ensuring robust performance across bacteria, fungi, viruses, and protozoa—providing a common basis for virulence gene identification.
(2) Cross-Pathogen Adaptability of Functional Module Mapping: Virulence modules defined in *E. coli* (effectors, adhesion, secretion, metabolic pathways) can be analogously mapped to other pathogen types. For example, fungal membrane proteins, viral capsid proteins, and protozoan secretory factors may correspond to similar functional categories.
(3) Enhanced Predictive Power via High-Dimensional Tools: Techniques such as AlphaFold-Multimer (AFM), protein language models (e.g., ESM), and GWAS enable structural inference and semantic embedding for candidate identification, even in contexts lacking homologs, exhibiting lifecycle divergence, or complex genome structures—thus improving generalization capacity.

The virulence factor mining and annotation strategy established for *E. coli* represents a scalable methodological paradigm extendable to diverse intestinal pathogens (fungi, parasites, viruses, prions). This framework provides critical support for broad-spectrum pathogen detection and control, advancing both basic research and translational applications in infectious disease management.

## 4 Molecular diagnostics informed by evolutionary Signatures: integrative strategies and practical challenges

Genomic variants accumulated during the long-term evolution of enteric pathogens reflect adaptive mechanisms underlying host colonization, virulence enhancement, and immune evasion. Concurrently, these variants provide critical molecular targets for precise diagnostics. Virulence genes, resistance determinants, and insertion sequences—characterized by lineage-specific stability and functional specificity—have emerged as core biomarkers for pathogen detection. Advances in large-scale genome sequencing have enabled the identification of evolutionarily representative genomic regions, facilitating their translation into rapid clinical diagnostic assays. Building on the comprehensive annotation of 355 *Escherichia coli* (*E. coli*) virulence factors, this section extends the “evolution-driven marker emergence” paradigm to fungal, parasitic, viral, and prion pathogens, emphasizing their diagnostic relevance across nucleic acid detection, protein conformational analysis, and related molecular modalities.

### 4.1 Diagnostic targets derived from pathogen genome Evolution: types and representative examples

Pathogen genomes acquire virulence determinants, resistance traits, and environmental adaptation features via horizontal gene transfer (HGT), recombination, point mutations, and chromosomal rearrangements, enhancing ecological fitness and pathogenicity (Good et al., 2025). These evolution-driven genetic variations facilitate niche expansion and virulence enhancement while serving as stable, functionally explicit biomarkers for molecular diagnostics. Key targets include virulence genes, resistance loci, pathogenicity islands, antigenic variation-related regions, and regulatory domains—all exhibiting high evolutionary conservation and functional specificity.

*E. coli* exemplifies these evolutionary dynamics. The O104:H4 strain, which acquired virulence elements from both uropathogenic (UPEC) and enterohemorrhagic (EHEC) lineages via HGT, harbors the stx2-encoding prophage and locus of enterocyte effacement (LEE) pathogenicity island—both characterized by high structural conservation and stable maintenance. These features render them critical targets for CRISPR-based diagnostics targeting persistent public health threats (Kimata et al., 2020). The high-risk sequence type 131 (ST131) clone, which coharbors β-lactamase genes (e.g., *bla*_CTX − M−15_) and virulence factors (e.g., aec, bap), facilitates global dissemination and multidrug resistance. Its genetic stability supports application in rapid field detection platforms such as loop-mediated isothermal amplification–lateral flow assay (LAMP-LFA; Mills et al., 2022). Furthermore, the highly conserved eae and tir genes within the LEE island, whose subtype distributions correlate tightly with serotypes, with structural variations localized to intergenic and terminal regions, serve as essential markers for multi-omics and machine learning (ML)–based strain typing and pathogenicity prediction (Sváb et al., 2022).

Pathogenicity in fungi primarily arises from chromosomal rearrangements and expansions of surface protein families. For example, *Candida auris* harbors 30 tandem repeats of the ALS4 gene (encoding 1844 amino acids) in the subtelomeric region of chromosome 5, augmenting adhesion and biofilm formation. The pronounced expression (~400-fold) and copy number variation (CNV) at this locus represent key targets for protein microarrays and ML approaches, enabling strain tracking and outbreak risk assessment in clinical settings (Bing et al., 2023).

Antigenic variation mediated by Variant Surface Protein (VSP) families is a hallmark of parasitic adaptation. In *Giardia lamblia*, hundreds of VSP genes undergo tightly regulated epigenetic switching to ensure monoallelic expression, facilitating immune evasion. This mechanism underpins pathogenic evolution and offers novel targets for structural bioinformatics and CRISPR-based interventions (Rodriguez-Walker et al., 2022).

RNA viruses exhibit rapid antigenic evolution driven by high mutation and recombination rates. In *Severe Acute Respiratory Syndrome Coronavirus 2* (SARS-CoV-2), mutations in the Spike protein's receptor-binding domain (RBD) and N-terminal domain (NTD)—notably N501Y and N439R—significantly enhance transmissibility and immune escape. Similarly, norovirus GII.4's major capsid protein VP1 contains hypervariable antigenic sites that facilitate immune evasion and epidemic spread. With over one million SARS-CoV-2 genomes sequenced, deep sequencing enables real-time variant monitoring and supports extensive ML modeling efforts (Harvey et al., 2021).

Prions, lacking nucleic acids, depend on conformational alterations of the prion protein (PrP) for pathogenicity. Polymorphisms in the PRNP gene modulate the stability of the β2-α2 loop and α3 helix in cellular PrP (PrP^∧^C), influencing susceptibility, transmission, and progression of chronic wasting disease (CWD). *In vitro* studies using protein misfolding cyclic amplification (PMCA) and nuclear magnetic resonance (NMR) have elucidated these polymorphisms' mechanistic roles in conversion to the scrapie isoform (PrP^∧^Sc), providing a basis for proteomic interventions and early diagnostic markers (Moazami-Goudarzi et al., 2021).

Diagnostic targets derived from pathogen evolutionary signatures encapsulate fundamental mechanisms of host adaptation and serve as a foundation for developing highly specific and stable molecular assays. Integration with multi-omics data and machine intelligence enhances detection efficiency of pathogen evolutionary dynamics, advancing precision medicine and public health surveillance.

### 4.2 Adaptation mechanisms between technological platforms and Biomarkers: from detection to imaging

Pathogen genome evolution not only drives the emergence of novel virulence and resistance mechanisms but also shapes recognizable molecular diagnostic targets. Diverse evolutionary products—including point mutations, genomic island insertions, and structural rearrangements—align with optimal detection technologies. This section categorizes major diagnostic platforms by biomarker type and systematically reviews their target adaptation mechanisms, with practical value illustrated through representative pathogens.

#### 4.2.1 Point mutations and minor variants: CRISPR-Cas and digital PCR systems

Point mutations represent pivotal adaptive mechanisms in pathogens, frequently linked to enhanced resistance or immune evasion. For example, non-synonymous mutations in the *mgrB* gene of *Klebsiella pneumoniae* (K. pneumoniae) inactivate the PhoP/PhoQ two-component regulatory system, upregulating lipid A modification genes that increase L-Ara4N or P-EtN modifications. These changes reduce colistin affinity, inducing resistance. Atomic force microscopy (AFM) and transmission electron microscopy (TEM) confirm that mutant strains exhibit thickened capsules and enhanced cell wall rigidity, which hinder antibiotic penetration. Increased capsular polysaccharide (CPS) synthesis and ion transport particle accumulation further amplify resistance and transmission potential, necessitating targeted detection and combination therapies (Yap et al., 2022).

CRISPR-Cas12a/Cas13a systems, with base-level recognition capability, enable precise CRISPR RNA (crRNA)-guided targeting of mutation sites (e.g., *stx2a*, *bla*_NDM − 1_) when combined with polymerase chain reaction (PCR) or recombinase polymerase amplification (RPA). This achieves visual detection of virulence and resistance mutations within 60 min. Optimized reverse transcription recombinase-aided amplification (RT-RAA)-Cas12a assays detect norovirus genotypes GII.4 and GII.17 at ultra-low viral loads (~0.1 copies/μL) within 30–40 min, offering rapid, portable field diagnostics (Qian et al., 2022).

Digital PCR (dPCR), with its absolute quantification capacity, sensitively detects resistance-related single nucleotide polymorphisms (SNPs; e.g., *rlmL, mltB*) in bloodstream infections. It facilitates low-frequency mutation screening and dynamic modeling, demonstrating superior sensitivity and specificity in diagnosing *Escherichia coli* (*E. coli*) bacteremia compared to conventional molecular methods. This positions dPCR as a promising novel rapid resistance biomarker (Kitagawa et al., 2025).

#### 4.2.2 Genomic islands and integrated elements: LAMP, RPA, and lateral flow assays

Pathogenicity islands or resistance plasmids acquired via horizontal transfer are key drivers of bacterial genome evolution. For instance, structural variations in the 3′ untranslated region (3′UTR) of *aggR* in enteroaggregative *E. coli* (EAEC) enhance transcript stability, upregulating AggR expression. This promotes motility, pAA plasmid conjugation, and adhesion-related metabolic pathways, significantly boosting pathogenicity. The locus of enterocyte effacement (LEE) island in enterohemorrhagic *E. coli* (EHEC) integrates at specific loci to encode virulence factors (e.g., *eae*), facilitating effector secretion and host attachment. EAEC's pAA plasmid coordinates adhesion and metabolism through *aggR* and *aafA*, intensifying virulence and plasmid dissemination (Prieto et al., 2021).

Due to their distinct structural specificity, these elements are well-suited for target recognition via loop-mediated isothermal amplification (LAMP) and RPA, combined with lateral flow assays (LFA) for equipment-free rapid onsite detection. LAMP-LFA systems demonstrate high sensitivity and adaptability in rapidly screening novel virulent strains (e.g., *E. coli* O104:H4, *Clostridioides difficile* ribotype 027 [RT027]) in complex samples, providing reliable technical support for food safety and public health surveillance (Nuchchanart et al., 2023).

#### 4.2.3 Structural rearrangements and repeat Expansions: nanopore sequencing and protein microarrays

Certain fungi and parasites exhibit structural rearrangements and copy number amplifications during evolution, necessitating high-throughput sequencing or structural probe techniques for detection. For example, multiple clinical strains of *Candida auris* acquire fluconazole resistance through duplication of *ERG11* and *TAC1B* genes, while *Giardia lamblia* achieves antigenic variation via tandem repeats and expression switching of Variant Surface Proteins (VSPs), underpinning chronic infection (Rodriguez-Walker et al., 2022). Such structural variants challenge conventional PCR and require long-read platforms like nanopore sequencing to resolve large rearrangements.

Structural prediction tools (e.g., AlphaFold) accurately model protein 3D conformations, aiding identification of domain expansions and diagnostic epitope variations, thereby enhancing conformational epitope recognition efficiency. Protein microarrays enable high-throughput screening of linear epitopes; when integrated with AlphaFold-mapped binding sites, they facilitate discovery of potential conformational epitopes, improving the specificity and accuracy of diagnostic targets (Grewal et al., 2024).

#### 4.2.4 Conformational changes and epigenetic modifications: aggregation detection and high-throughput mass spectrometry

Certain pathogen evolutionary variations manifest not at the sequence level but as protein conformational changes or post-translational modifications. For instance, in variant Creutzfeldt-Jakob Disease (vCJD), PrP^∧^Sc exhibits unique glycosylation patterns and amyloid aggregate conformations, leading to enhanced amplification efficiency and sensitivity in real-time quaking-induced conversion (RT-QuIC) and protein misfolding cyclic amplification (PMCA) assays. RT-QuIC monitors PrP^∧^Sc-induced fibril formation in real time, while PMCA amplifies PrP^∧^Sc via autocatalytic conversion, jointly supporting early non-invasive vCJD diagnosis (Camacho et al., 2019).

Fungi also commonly display species-specific glycosylation modifications with diagnostic potential. Clinical *Candida* isolates frequently present O-linked β-N-acetylglucosamine (O-GlcNAc) glycosylation on adhesion proteins, serving as candidate diagnostic antigens. Surface-enhanced Raman spectroscopy (SERS), combined with Fe_3_O_4_@polyethyleneimine (PEI) magnetic nanoparticle capture and silver nanoparticle (AgNP) signal enhancement, directly detects O-GlcNAc spectral features. Coupled with orthogonal partial least squares discriminant analysis (OPLS-DA) modeling, classification accuracy reaches up to 99.8%, outperforming culture-dependent matrix-assisted laser desorption/ionization time-of-flight mass spectrometry (MALDI-TOF MS). This non-destructive SERS strategy reduces detection time to 1 h, demonstrating significant advantages for early diagnosis of candidemia (Hu et al., 2021).

### 4.3 Trends in pan-pathogen diagnostics driven by multi-omics and intelligent computing

As gut pathogens evolve at the population level, traditional diagnostic methods reliant on single virulence or resistance factors have increasingly exposed their limitations, particularly in contexts of co-infection, frequent mutation, and enhanced environmental adaptation. Multi-omics strategies integrated with intelligent tools such as machine learning (ML) are driving the development of “cross-pathogen” diagnostic platforms, providing a systematic framework to unravel complex infectious etiologies.

Recent metagenomic sequencing combined with variant analysis has been widely used to monitor horizontal transfer of resistance islands and virulence factors within gut microbiomes. For example, metagenomic studies identified plasmid-mediated horizontal gene transfer (HGT) of CTX-M-type β-lactamase genes (*bla*_CTX − M_) from *Escherichia coli* (*E. coli*) to *Klebsiella pneumoniae* (*K. pneumoniae*) in hospital wastewater in northern India, with high abundance in urban hospital samples. The gene cassette structure of class 1 integrons (including *intI1* and *sul1*) co-occurs tightly with *bla*_CTX − M_, and specific domains such as the *attC* site are recognized as evolutionary targets in the HGT process. These findings underscore hospital wastewater as a critical reservoir for antibiotic resistance gene (ARG) dissemination and emphasize the need for intervention strategies targeting integron structures to curb antimicrobial resistance (AMR) spread (Talat et al., 2023).

Multi-omics approaches also demonstrate significant diagnostic value for viral and protozoan pathogens. Norovirus genotype GII.4 (NoV GII.4) enhances replication and antigenic variation through ORF1/2 recombination and mutations in the RNA-dependent RNA polymerase (RdRp) region, with key antigenic mutations in sites A and D of the P2 domain driving immune evasion. Omics analyses reveal these mutations cluster in the P2 domain, where machine learning models predict immune escape potential based on sequence and structural features. Consequently, strategies targeting conserved regions of the P2 domain for broad-spectrum antibodies and multivalent virus-like particle (VLP) vaccines have been proposed to address challenges posed by ongoing GII.4 evolution (Tohma et al., 2022).

In *Giardia lamblia* (*G. lamblia*), the Variant Surface Protein (VSP) family exhibits pronounced sequence polymorphism and splicing isoform diversity during host adaptation, resulting in highly heterogeneous expression profiles. Single-cell transcriptomics reveal dynamic VSP expression patterns underlying antigenic variation, and deep learning models trained on these data can automatically classify virulence subtypes and identify key features linked to immune escape and host specificity. This approach offers a powerful tool for rapid prediction of *Giardia* virulence evolution and individual adaptability, informing precise intervention strategies (Rodriguez-Walker et al., 2022).

Structural omics coupled with artificial intelligence (AI)-assisted prediction is emerging as a powerful tool to discover cross-species virulence domains. AlphaFold2 combined with sequence alignments has uncovered highly conserved β-helical core domains in the ALS family adhesins of *Candida albicans* (*C. albicans*) and *Candida tropicalis* (*C. tropicalis*), providing a common structural basis for cross-species adhesion functionality. This conserved domain serves as an ideal target for protein microarray typing, supporting lineage tracing and pathogenicity studies, and aiding the development of intervention strategies (Oh et al., 2021). Similar approaches have been employed to explore previously unannotated virulence factors in enterohemorrhagic *E. coli* (EHEC) strains. Studies use AlphaFold2 to predict their 3D structures and apply random forest algorithms to identify discriminative functional epitopes, enabling the design of novel multiplex diagnostic platforms that bridge “gene sequence annotation” and “structural-functional localization.”

With increasing complexity of evolutionary mutations and expansion of pathogen spectra, integration of multi-omics data with AI algorithms is driving diagnostics from “specific site recognition” toward “generalized evolutionary prediction.” By categorizing, aggregating, and mapping mutation features onto structure-function relationships, diagnostic systems are increasingly capable of identifying novel variants, predicting transmission potential, and forecasting resistance trends. This paradigm shift not only enhances diagnostic accuracy for complex infections but also provides critical technological support for rapid responses to emerging pathogens.

### 4.4 Technology integration and real-world challenges: from laboratory to clinic and field

Despite rapid advancements in molecular diagnostics due to their high sensitivity and specificity for infectious disease detection, translating these technologies from laboratory research to real-world field applications presents persistent practical constraints. Particularly given the rapid evolution and transmission diversity of gut pathogens, single detection techniques often fail to balance broad-spectrum capability, cost-effectiveness, and field adaptability. Consequently, integrated technological approaches have become essential for overcoming these barriers.

#### 4.4.1 Multi-technology integration: enhancing field adaptability

CRISPR-Cas systems, loop-mediated isothermal amplification (LAMP), and protein structural recognition technologies are increasingly integrated for synergistic application. For example, portable diagnostic platforms combining CRISPR/Cas12a with LAMP enable multiplex detection of *Escherichia coli* (*E. coli*) virulence factors within 40 min, providing instrument-free visual interpretation via lateral flow strips or LED blue light. This approach achieves high sensitivity and specificity suitable for field applications in resource-limited regions (Shi et al., 2022). Field validation across rural clinical settings demonstrated 96.4% detection sensitivity, confirming the utility of integrated platforms for pathogen screening in low-resource environments.

#### 4.4.2 Sample compatibility optimization: mitigating inhibitor interference

Complex matrices such as feces and wastewater contain inhibitors that compromise diagnostic accuracy through false-negative results. Incorporating reduced graphene oxide (rGO) into lateral flow assay (LFA) electrodes enhances electrochemical signal stability by over three-fold, leveraging rGO's electron transfer efficiency, gold nanoparticle immobilization capacity, and enzyme stabilization properties. This platform successfully identified Shiga toxin-positive samples in simulated diarrheal stool, demonstrating robust potential for field diagnostics under challenging sample conditions (Calucho et al., 2024).

#### 4.4.3 Addressing expression heterogeneity: from structural to functional monitoring

Virulence gene expression heterogeneity represents a critical gap in current surveillance. Hybridization chain reaction (HCR) RNA-fluorescence *in situ* hybridization (RNA-FISH) revealed spatiotemporal variation in *Candida albicans* (C. albicans) *HWP1* expression during murine tongue mucosa infection, illustrating dynamic virulence regulation under host immune pressure. This phenomenon may explain asymptomatic carriers or atypical presentations, indicating that future field diagnostics should incorporate expression-sensitive biomarkers (Lindemann-Perez et al., 2024).

#### 4.4.4 Translational framework: bidirectional adaptation

Effective translation from laboratory validation to field deployment requires aligning diagnostic design with pathogen evolutionary mechanisms. Prioritizing stress-resistant conserved domains avoids overreliance on variable promoter targets. Concurrently, integrating virulence expression prediction into diagnostic models enhances interpretability for heterogeneous samples and complex infections.

This bidirectional integration model—synthesizing evolutionary insights with technological innovation—provides a critical pathway toward precise, efficient, and equitable infectious disease management.

## 5 Conclusions and future perspectives

The genomic evolution of enteric pathogens continually poses global public health challenges by driving enhanced virulence, dissemination of antimicrobial resistance (AMR), and host adaptation. Dynamic mechanisms—including horizontal gene transfer (HGT), chromosomal rearrangements, hypermutation, and epigenetic regulation—critically modulate pathogenic potential and epidemiology, thereby influencing the global burden of infectious diseases and intervention strategies. This review systematically examines the evolutionary trajectories of five major enteric pathogen classes (bacteria, fungi, parasites, viruses, and prions), emphasizing the central roles of virulence gene clusters, resistance elements, and mobile genetic elements (MGEs) in microbial pathogenicity. Using *Escherichia coli* (*E. coli*) as a paradigmatic model, we performed a comprehensive multidimensional annotation of 335 virulence factors (Figure 1), validating the feasibility of a closed-loop framework integrating genomic evolution, biomarker identification, and diagnostic assay development. This framework elucidates key molecular principles underlying pathogen-host interactions and provides methodological support for advancing precision diagnostics.

Recent advances highlight the translational potential of evolutionarily conserved diagnostic targets: CRISPR-Cas systems targeting the *stx2* gene achieved complete concordance with clinical outcomes; loop-mediated isothermal amplification combined with lateral flow assay (LAMP-LFA) demonstrated 98.7% sensitivity for detecting *bla*_NDM − 1_ resistance mutations in fecal specimens; and AlphaFold-based structural domain prediction successfully identified immunogenic regions of the *Cryptococcus HWP1* protein. These findings validate the robustness of evolutionarily stable biomarkers and provide a data-driven foundation for iterative technological refinement.

Nonetheless, the inherent complexity of enteric pathogen evolution poses significant challenges for diagnostic development:

(1) Phenotypic plasticity arising from host microenvironmental dynamics—including low-abundance co-infections and stress-induced gene expression reprogramming—limits the reliability of single-target assays;
(2) Rapid HGT of resistance elements outpaces diagnostic update cycles, necessitating dynamic surveillance frameworks; and
(3) Resource constraints in low-income regions restrict deployment of advanced technologies.

Future research directions to address these challenges include:

(1) Integration of multi-omics and real-time monitoring approaches: Leveraging single-cell spatial transcriptomics, deep mutational scanning, and nanopore sequencing to construct “digital twin” models that capture pathogen virulence heterogeneity and resistance mutation trajectories.
(2) Development of cost-effective, high-sensitivity diagnostics: Utilizing biomimetic magnetic bead capture, reduced graphene oxide (rGO)-quantum dot composite probes, and smartphone-based imaging to realize paper-based microfluidic platforms for point-of-care testing with per-assay costs under $5.
(3) Establishment of “One Health” surveillance networks: Integrating cloud-based artificial intelligence (AI) and real-time sequencing data across human, animal, and environmental reservoirs to enable early warning of emerging cross-species pathogens such as chronic wasting disease (CWD) prions.

In conclusion, elucidating genomic evolution in enteric pathogens not only advances fundamental understanding of disease mechanisms but also drives innovation in diagnostic development and public health strategies. The closed-loop paradigm of evolution-driven biomarker discovery and diagnostic translation holds promise for shifting infectious disease control from reactive responses toward proactive surveillance and precision intervention on a global scale.

## References

[B1] Arias-Agudelo L. M. Garcia-Montoya G. Cabarcas F. Galvan-Diaz A. L. Alzate J. F. (2020). Comparative genomic analysis of the principal *Cryptosporidium* species that infect humans. PeerJ 8:e10478. doi: 10.7717/peerj.10478 33344091 PMC7718795

[B2] Aurrecoechea C. Brestelli J. Brunk B. P. Fischer S. Gajria B. Gao X. . (2010). EuPathDB: a portal to eukaryotic pathogen databases. Nucleic Acids Res. 38, D415–D419. doi: 10.1093/nar/gkp941 19914931 PMC2808945

[B3] Bakkeren E. Gul E. Huisman J. S. Steiger Y. Rocker A. Hardt W. D. . (2022). Impact of horizontal gene transfer on emergence and stability of cooperative virulence in Salmonella Typhimurium. Nat. Commun. 13:1939. doi: 10.1038/s41467-022-29597-7 35410999 PMC9001671

[B4] Bing J. Guan Z. Zheng T. Zhang Z. Fan S. Ennis C. L. . (2023). Clinical isolates of *Candida auris* with enhanced adherence and biofilm formation due to genomic amplification of ALS4. PLoS Pathog. 19:e1011239. doi: 10.1371/journal.ppat.1011239 36913408 PMC10035925

[B5] Blum M. Andreeva A. Florentino L. C. Chuguransky S. R. Grego T. Hobbs E. . (2025). InterPro: the protein sequence classification resource in 2025. Nucleic Acids Res. 53, D444–D456. doi: 10.1093/nar/gkae1082 39565202 PMC11701551

[B6] Calucho E. Alvarez-Diduk R. Piper A. Rossetti M. Nevanen T. K. Merkoci A. . (2024). Reduced graphene oxide electrodes meet lateral flow assays: a promising path to advanced point-of-care diagnostics. Biosens. Bioelectron. 258:116315. doi: 10.1016/j.bios.2024.116315 38701536

[B7] Camacho M. V. Telling G. Kong Q. Gambetti P. Notari S. (2019). Role of prion protein glycosylation in replication of human prions by protein misfolding cyclic amplification. Lab. Invest. 99, 1741–1748. doi: 10.1038/s41374-019-0282-1 31249376

[B8] Cannon J. L. Bonifacio J. Bucardo F. Buesa J. Bruggink L. Chan M. C. . (2021). Global trends in norovirus genotype distribution among children with acute gastroenteritis. Emerg. Infect. Dis. 27, 1438–1445. doi: 10.3201/eid2705.204756 33900173 PMC8084493

[B9] Carlson K. B. Dilley A. O'Grady T. Johnson J. A. Lopman B. Viscidi E. . (2024). A narrative review of norovirus epidemiology, biology, and challenges to vaccine development. NPJ Vaccines 9:94. doi: 10.1038/s41541-024-00884-2 38811605 PMC11137017

[B10] Cavas L. Kirkiz I. (2022). Characterization of siderophores from *Escherichia coli* strains through genome mining tools: an antiSMASH study. AMB Express 12:74. doi: 10.1186/s13568-022-01421-x 35704153 PMC9200922

[B11] Chhabra P. Tully D. C. Mans J. Niendorf S. Barclay L. Cannon J. L. . (2024). Emergence of novel norovirus GII.4 variant. Emerg. Infect. Dis. 30, 163–167. doi: 10.3201/eid3001.231003 38063078 PMC10756382

[B12] Collaborators G. B. D. A. R. (2024). Global burden of bacterial antimicrobial resistance 1990-2021: a systematic analysis with forecasts to 2050. Lancet 404, 1199–1226. doi: 10.1016/S0140-6736(24)01867-1 39299261 PMC11718157

[B13] Collaborators G. B. D. D. D. (2025). Global, regional, and national age-sex-specific burden of diarrhoeal diseases, their risk factors, and aetiologies, 1990-2021, for 204 countries and territories: a systematic analysis for the Global Burden of Disease Study 2021. Lancet Infect. Dis. 25, 519–536. doi: 10.1016/S1473-3099(24)00691-1 39708822 PMC12018300

[B14] Dai L. Wu Z. Sahin O. Zhao S. Yu E. W. Zhang Q. . (2024). Mutation-based mechanism and evolution of the potent multidrug efflux pump RE-CmeABC in Campylobacter. Proc. Natl. Acad. Sci. U.S.A. 121:e2415823121. doi: 10.1073/pnas.2415823121 39602248 PMC11665921

[B15] Duque Velasquez C. Kim C. Haldiman T. Kim C. Herbst A. Aiken J. . (2020). Chronic wasting disease (CWD) prion strains evolve via adaptive diversification of conformers in hosts expressing prion protein polymorphisms. J. Biol. Chem. 295, 4985–5001. doi: 10.1074/jbc.RA120.012546 32111742 PMC7152757

[B16] El-Heneidy A. Grimwood K. Lambert S. B. Ware R. S. (2023). Interference between enteric viruses and live-attenuated rotavirus vaccine virus in a healthy Australian Birth Cohort. J. Infect. Dis. 228, 851–856. doi: 10.1093/infdis/jiad094 37014728 PMC10547457

[B17] Elshobary M. E. Badawy N. K. Ashraf Y. Zatioun A. A. Masriya H. H. Ammar M. M. . (2025). Combating antibiotic resistance: mechanisms, multidrug-resistant pathogens, and novel therapeutic approaches: an updated review. Pharmaceuticals 18:402. doi: 10.3390/ph18030402 40143178 PMC11944582

[B18] Feng J. M. Yang C. L. Tian H. F. Wang J. X. Wen J. F. (2020). Identification and evolutionary analysis of the nucleolar proteome of *Giardia lamblia*. BMC Genom. 21:269. doi: 10.1186/s12864-020-6679-9 32228450 PMC7104513

[B19] Frazao N. Sousa A. Lassig M. Gordo I. (2019). Horizontal gene transfer overrides mutation in *Escherichia coli* colonizing the mammalian gut. Proc. Natl. Acad. Sci. U.S.A. 116, 17906–17915. doi: 10.1073/pnas.1906958116 31431529 PMC6731689

[B20] Galperin M. Y. Wolf Y. I. Makarova K. S. Vera Alvarez R. Landsman D. Koonin E. V. (2021). COG database update: focus on microbial diversity, model organisms, and widespread pathogens. Nucleic Acids Res. 49, D274–D281. doi: 10.1093/nar/gkaa1018 33167031 PMC7778934

[B21] Garud N. R. Good B. H. Hallatschek O. Pollard K. S. (2019). Evolutionary dynamics of bacteria in the gut microbiome within and across hosts. PLoS Biol. 17:e3000102. doi: 10.1371/journal.pbio.3000102 30673701 PMC6361464

[B22] Gelpi E. Baiardi S. Nos C. Dellavalle S. Aldecoa I. Ruiz-Garcia R. . (2022). Sporadic Creutzfeldt-Jakob disease VM1: phenotypic and molecular characterization of a novel subtype of human prion disease. Acta Neuropathol. Commun. 10:114. doi: 10.1186/s40478-022-01415-7 35978418 PMC9387077

[B23] Geurtsen J. de Been M. Weerdenburg E. Zomer A. McNally A. Poolman J. . (2022). Genomics and pathotypes of the many faces of *Escherichia coli*. FEMS Microbiol. Rev. 46:fuac031. doi: 10.1093/femsre/fuac031 35749579 PMC9629502

[B24] Good B. H. Bhatt A. S. McDonald M. J. (2025). Unraveling the tempo and mode of horizontal gene transfer in bacteria. Trends Microbiol. 33, 853–865. doi: 10.1016/j.tim.2025.03.009 40274494 PMC13244654

[B25] Greig D. R. Jenkins C. Dallman T. J. (2020). A shiga toxin-encoding prophage recombination event confounds the phylogenetic relationship between two isolates of *Escherichia coli* O157:H7 from the same patient. Front. Microbiol. 11:588769. doi: 10.3389/fmicb.2020.588769 33193248 PMC7645076

[B26] Grewal S. Hegde N. Yanow S. K. (2024). Integrating machine learning to advance epitope mapping. Front. Immunol. 15:1463931. doi: 10.3389/fimmu.2024.1463931 39403389 PMC11471525

[B27] Group I. P. C. (2024). Global burden associated with 85 pathogens in 2019: a systematic analysis for the Global Burden of Disease Study 2019. Lancet Infect. Dis. 24, 868–895. doi: 10.1016/S1473-3099(24)00158-0 38640940 PMC11269650

[B28] Haese-Hill W. Crouch K. Otto T. D. (2024). Annotation and visualization of parasite, fungi and arthropod genomes with companion. Nucleic Acids Res. 52, W39–W44. doi: 10.1093/nar/gkae378 38752499 PMC11223846

[B29] Harbi D. Parthiban M. Gendoo D. M. Ehsani S. Kumar M. Schmitt-Ulms G. . (2012). PrionHome: a database of prions and other sequences relevant to prion phenomena. PLoS ONE 7:e31785. doi: 10.1371/journal.pone.0031785 22363733 PMC3282748

[B30] Harvey W. T. Carabelli A. M. Jackson B. Gupta R. K. Thomson E. C. Harrison E. M. . (2021). SARS-CoV-2 variants, spike mutations and immune escape. Nat. Rev. Microbiol. 19, 409–424. doi: 10.1038/s41579-021-00573-0 34075212 PMC8167834

[B31] Hawkey J. Monk J. M. Billman-Jacobe H. Palsson B. Holt K. E. (2020). Impact of insertion sequences on convergent evolution of Shigella species. PLoS Genet. 16:e1008931. doi: 10.1371/journal.pgen.1008931 32644999 PMC7373316

[B32] Homma F. Huang J. van der Hoorn R. A. L. (2023). AlphaFold-Multimer predicts cross-kingdom interactions at the plant-pathogen interface. Nat. Commun. 14:6040. doi: 10.1038/s41467-023-41721-9 37758696 PMC10533508

[B33] Hoxie I. Dennehy J. J. (2020). Intragenic recombination influences rotavirus diversity and evolution. Virus Evol. 6:vez059. doi: 10.1093/ve/vez059 31949920 PMC6955627

[B34] Hu S. Kang H. Gu F. Wang C. Cheng S. Gong W. . (2021). Rapid detection method for pathogenic candida captured by magnetic nanoparticles and identified using SERS via AgNPs(). Int. J. Nanomed. 16, 941–950. doi: 10.2147/IJN.S285339 33603361 PMC7884937

[B35] Huang Y. Wang Y. Huang X. Yu X. (2024). Unveiling the overlooked fungi: the vital of gut fungi in inflammatory bowel disease and colorectal cancer. Gut Pathog. 16:59. doi: 10.1186/s13099-024-00651-7 39407244 PMC11481806

[B36] Ibe C. Pohl C. H. (2024). Update on the structure and function of *Candida albicans* drug efflux protein, Cdr1. Fungal Genet. Biol. 175:103938. doi: 10.1016/j.fgb.2024.103938 39486613

[B37] Islam M. M. Mahbub N. U. Hong S. T. Chung H. J. (2024). Gut bacteria: an etiological agent in human pathological conditions. Front. Cell. Infect. Microbiol. 14:1291148. doi: 10.3389/fcimb.2024.1291148 39439902 PMC11493637

[B38] Jankovska N. Rusina R. Bruzova M. Parobkova E. Olejar T. Matej R. . (2021). Human prion disorders: review of the current literature and a twenty-year experience of the national surveillance center in the Czech Republic. Diagnostics 11:1821. doi: 10.3390/diagnostics11101821 34679519 PMC8534461

[B39] Jin Z. Sato Y. Kawashima M. Kanehisa M. (2023). KEGG tools for classification and analysis of viral proteins. Protein Sci. 32:e4820. doi: 10.1002/pro.4820 37881892 PMC10661063

[B40] Johansen R. L. Schouw C. H. Madsen T. V. Nielsen X. C. Engberg J. (2023). Epidemiology of gastrointestinal infections: lessons learned from syndromic testing, Region Zealand, Denmark. Eur. J. Clin. Microbiol. Infect. Dis. 42, 1091–1101. doi: 10.1007/s10096-023-04642-5 37468662 PMC10427544

[B41] Kakade P. Sircaik S. Maufrais C. Ene I. V. Bennett R. J. (2023). Aneuploidy and gene dosage regulate filamentation and host colonization by *Candida albicans*. Proc. Natl. Acad. Sci. U.S.A. 120:e2218163120. doi: 10.1073/pnas.2218163120 36893271 PMC10089209

[B42] Karamati S. A. Mirjalali H. Niyyati M. Yadegar A. Asadzadeh Aghdaei H. Haghighi A. . (2021). Association of blastocystis ST6 with higher protease activity among symptomatic subjects. BMC Microbiol. 21:285. doi: 10.1186/s12866-021-02341-9 34666703 PMC8524833

[B43] Kaur J. Nobile C. J. (2023). Antifungal drug-resistance mechanisms in *Candida biofilms*. Curr. Opin. Microbiol. 71:102237. doi: 10.1016/j.mib.2022.102237 36436326 PMC11569868

[B44] Kimata K. Lee K. Watahiki M. Isobe J. Ohnishi M. Iyoda S. . (2020). Global distribution of epidemic-related Shiga toxin 2 encoding phages among enteroaggregative *Escherichia coli*. Sci. Rep. 10:11738. doi: 10.1038/s41598-020-68462-9 32678145 PMC7366661

[B45] Kistler K. E. Bedford T. (2023). An atlas of continuous adaptive evolution in endemic human viruses. Cell Host Microbe 31, 1898–1909. doi: 10.1016/j.chom.2023.09.012 37883977 PMC12129310

[B46] Kitagawa H. Kojima M. Tadera K. Kogasaki S. Omori K. Nomura T. . (2025). Clinical diagnostic performance of droplet digital PCR for pathogen detection in patients with *Escherichia coli* bloodstream infection: a prospective observational study. BMC Infect. Dis. 25:22. doi: 10.1186/s12879-024-10396-y 39757158 PMC11702014

[B47] Lau W. Y. V. Taylor P. K. Brinkman F. S. L. Lee A. H. Y. (2023). Pathogen-associated gene discovery workflows for novel antivirulence therapeutic development. EBioMed. 88:104429. doi: 10.1016/j.ebiom.2022.104429 36628845 PMC9843249

[B48] Lee Y. Robbins N. Cowen L. E. (2023). Molecular mechanisms governing antifungal drug resistance. NPJ Antimicrob. Resist. 1:5. doi: 10.1038/s44259-023-00007-2 38686214 PMC11057204

[B49] Li X. Wang J. Zhang Y. Zhao Y. Shi Y. (2025). Evolutionary characterization and pathogenicity of the highly virulent human-porcine reassortant G9P[23] porcine rotavirus HB05 strain in several Chinese provinces. Front. Microbiol. 16:1539905. doi: 10.3389/fmicb.2025.1539905 40160270 PMC11949960

[B50] Lindemann-Perez E. Rodriguez D. L. Pérez J. C. (2024). An approach to analyze spatiotemporal patterns of gene expression at single- cell resolution in Candida albicans-infected mouse tongues. mSphere. 9:e0028224. doi: 10.1128/msphere.00282-24 39171917 PMC11423565

[B51] Liu B. Zheng D. Zhou S. Chen L. Yang J. (2022). VFDB 2022: a general classification scheme for bacterial virulence factors. Nucleic Acids Res. 50, D912–D917. doi: 10.1093/nar/gkab1107 34850947 PMC8728188

[B52] Liu X. Wang M. Li S. Li J. Xiao J. Li H. . (2022). Genomic and evolutionary characteristics of G9P[8], the dominant group a rotavirus in China (2016-2018). Front. Microbiol. 13:997957. doi: 10.3389/fmicb.2022.997957 36187963 PMC9522900

[B53] Losa M. Morsy Y. Emmenegger M. Manz S. M. Schwarz P. Aguzzi A. . (2024). Longitudinal microbiome investigation throughout prion disease course reveals pre- and symptomatic compositional perturbations linked to short-chain fatty acid metabolism and cognitive impairment in mice. Front. Microbiol. 15:1412765. doi: 10.3389/fmicb.2024.1412765 38919500 PMC11196846

[B54] Ludwig-Begall L. F. Mauroy A. Thiry E. (2021). Noroviruses-the state of the art, nearly fifty years after their initial discovery. Viruses 13:1541. doi: 10.3390/v13081541 34452406 PMC8402810

[B55] Mahbub N. U. Islam M. M. Hong S. T. Chung H. J. (2024). Dysbiosis of the gut microbiota and its effect on alpha-synuclein and prion protein misfolding: consequences for neurodegeneration. Front. Cell. Infect. Microbiol. 14:1348279. doi: 10.3389/fcimb.2024.1348279 38435303 PMC10904658

[B56] Marcos-Vilchis A. Espinosa N. Alvarez A. F. Puente J. L. Soto J. E. González-Pedrajo B. . (2025). On the role of the sorting platform in hierarchical type III secretion regulation in enteropathogenic *Escherichia coli*. J. Bacteriol. 207, e00446–e00424. doi: 10.1128/jb.00446-24 40029102 PMC11925242

[B57] Marie C. Petri W. A. Jr. (2014). Regulation of virulence of *Entamoeba histolytica*. Annu. Rev. Microbiol. 68, 493–520. doi: 10.1146/annurev-micro-091313-103550 25002094 PMC9006484

[B58] Mhango C. Banda A. Chinyama E. Mandolo J. J. Kumwenda O. Malamba-Banda C. . (2023). Comparative whole genome analysis reveals re-emergence of human Wa-like and DS-1-like G3 rotaviruses after Rotarix vaccine introduction in Malawi. Virus Evol. 9:vead030. doi: 10.1093/ve/vead030 37305707 PMC10256189

[B59] Mills E. G. Martin M. J. Luo T. L. Ong A. C. Maybank R. Corey B. W. . (2022). A one-year genomic investigation of *Escherichia coli* epidemiology and nosocomial spread at a large US healthcare network. Genome Med. 14:147. doi: 10.1186/s13073-022-01150-7 36585742 PMC9801656

[B60] Moazami-Goudarzi K. Andréoletti O. Vilotte J.-L. Béringue V. (2021). Review on PRNP genetics and susceptibility to chronic wasting disease of Cervidae. Vet. Res. 52:128. doi: 10.1186/s13567-021-00993-z 34620247 PMC8499490

[B61] Nasrollahian S. Graham J. P. Halaji M. (2024). A review of the mechanisms that confer antibiotic resistance in pathotypes of *E. coli*. Front. Cell. Infect. Microbiol. 14:1387497. doi: 10.3389/fcimb.2024.1387497 38638826 PMC11024256

[B62] Nuchchanart W. Pikoolkhao P. Saengthongpinit C. (2023). Development of a lateral flow dipstick test for the detection of 4 strains of *Salmonella* spp. in animal products and animal production environmental samples based on loop-mediated isothermal amplification. Anim. Biosci. 36, 654–670. doi: 10.5713/ab.22.0151 36108678 PMC9996269

[B63] Oh S. H. Schliep K. Isenhower A. Rodriguez-Bobadilla R. Vuong V. M. Fields C. J. . (2021). Using genomics to shape the definition of the agglutinin-like sequence (ALS) family in the saccharomycetales. Front. Cell. Infect. Microbiol. 11:794529. doi: 10.3389/fcimb.2021.794529 34970511 PMC8712946

[B64] Okumu N. O. Muloi D. M. Moodley A. Watson J. Kiarie A. Ochieng L. . (2025). Antimicrobial resistance in community-acquired enteric pathogens among children aged < /= 10-years in low-and middle-income countries: a systematic review and meta-analysis. Front. Microbiol. 16:1539160. doi: 10.3389/fmicb.2025.1539160 40356650 PMC12066647

[B65] Pakbin B. Bruck W. M. Rossen J. W. A. (2021). Virulence factors of enteric pathogenic *Escherichia coli*: a review. Int. J. Mol. Sci. 22:9922. doi: 10.3390/ijms22189922 34576083 PMC8468683

[B66] Prieto A. Bernabeu M. Sanchez-Herrero J. F. Perez-Bosque A. Miro L. Bauerl C. . (2021). Modulation of AggR levels reveals features of virulence regulation in enteroaggregative *E. coli*. Commun. Biol. 4:1295. doi: 10.1038/s42003-021-02820-9 34785760 PMC8595720

[B67] Qian W. Huang J. Wang T. Fan C. Kang J. Zhang Q. . (2022). Ultrasensitive and visual detection of human norovirus genotype GII.4 or GII.17 using CRISPR-Cas12a assay. Virol. J. 19:150. doi: 10.1186/s12985-022-01878-z 36115975 PMC9482751

[B68] Quan Y. Zhang K. X. Zhang H. Y. (2023). The gut microbiota links disease to human genome evolution. Trends Genet. 39, 451–461. doi: 10.1016/j.tig.2023.02.006 36872184

[B69] Roach S. N. Langlois R. A. (2021). Intra- and cross-species transmission of astroviruses. Viruses 13:1127. doi: 10.3390/v13061127 34208242 PMC8230745

[B70] Rodriguez-Walker M. Molina C. R. Lujan L. A. Saura A. Jerlstrom-Hultqvist J. Svard S. G. . (2022). Comprehensive characterization of Cysteine-rich protein-coding genes of *Giardia lamblia* and their role during antigenic variation. Genomics 114:110462. doi: 10.1016/j.ygeno.2022.110462 35998788

[B71] Scott T. A. Baker K. S. Trotter C. Jenkins C. Mostowy S. Hawkey J. . (2025). Shigella sonnei: epidemiology, evolution, pathogenesis, resistance and host interactions. Nat. Rev. Microbiol. 23, 303–317. doi: 10.1038/s41579-024-01126-x 39604656

[B72] Sharma N. Das A. Nair A. V. Sethi P. Negi V. D. Chakravortty D. . (2024). CRISPR-Cas system positively regulates virulence of *Salmonella enterica* serovar Typhimurium. Gut Pathog. 16:63. doi: 10.1186/s13099-024-00653-5 39462402 PMC11514906

[B73] Shears R. K. Grencis R. K. (2022). Whipworm secretions and their roles in host-parasite interactions. Parasites Vectors 15:348. doi: 10.1186/s13071-022-05483-5 36175934 PMC9524059

[B74] Shi Y. Kang L. Mu R. Xu M. Duan X. Li Y. . (2022). CRISPR/Cas12a-enhanced loop-mediated isothermal amplification for the visual detection of *Shigella flexneri*. Front. Bioeng. Biotechnol. 10:845688. doi: 10.3389/fbioe.2022.845688 35265606 PMC8899461

[B75] Smoak R. A. Snyder L. F. Fassler J. S. He B. Z. (2023). Parallel expansion and divergence of an adhesin family in pathogenic yeasts. Genetics 223:iyad024. doi: 10.1093/genetics/iyad024 36794645 PMC10319987

[B76] Steele-Ogus M. C. Obenaus A. M. Sniadecki N. J. Paredez A. R. (2022). Disc and actin associated protein 1 influences attachment in the intestinal parasite *Giardia lamblia*. PLoS Pathog. 18:e1010433. doi: 10.1371/journal.ppat.1010433 35333908 PMC8986099

[B77] Sváb D. Falgenhauer L. Mag T. Chakraborty T. Tóth I. (2022). Genomic diversity, virulence gene, and prophage arrays of bovine and human shiga toxigenic and enteropathogenic *Escherichia coli* strains isolated in hungary. Front. Microbiol. 13:896296. doi: 10.3389/fmicb.2022.896296 35865933 PMC9294531

[B78] Talat A. Blake K. S. Dantas G. Khan A. A.-O. (2023). Metagenomic insight into microbiome and antibiotic resistance genes of high clinical concern in urban and rural hospital wastewater of northern india origin: a major reservoir of antimicrobial resistance. Microbiol Spectr. 11:e0410222. doi: 10.1128/spectrum.04102-22 36786639 PMC10100738

[B79] Todd R. T. Wikoff T. D. Forche A. Selmecki A. (2019). Genome plasticity in *Candida albicans* is driven by long repeat sequences. Elife. 8:e45954. doi: 10.7554/eLife.45954 31172944 PMC6591007

[B80] Tohma K. Ford-Siltz L. A. Kendra J. A. Parra G. I. (2022). Dynamic immunodominance hierarchy of neutralizing antibody responses to evolving GII.4 noroviruses. Cell Rep. 39:110689. doi: 10.1016/j.celrep.2022.110689 35417705

[B81] Urban M. Cuzick A. Seager J. Wood V. Rutherford K. Venkatesh Shilpa Y. . (2022). PHI-base in 2022: a multi-species phenotype database for pathogen–host interactions. Nucleic Acids Res. 50, D837–D47. doi: 10.1093/nar/gkab1037 34788826 PMC8728202

[B82] Vallabhaneni S. Mody R. K. Walker T. Chiller T. (2016). The global burden of fungal diseases. Infect. Dis. Clin. North Am. 30, 1–11. doi: 10.1016/j.idc.2015.10.004 26739604

[B83] Voorhies M. Cohen S. Shea T. Petrus S. Muñoz J. F. Poplawski S. . (2021). Chromosome-level genome assembly of a human fungal pathogen reveals synteny among geographically distinct species. bioRxiv. doi: 10.1101/2021.07.13.452254 35089059 PMC8725592

[B84] Wallace M. J. Fishbein S. R. S. Dantas G. (2020). Antimicrobial resistance in enteric bacteria: current state and next-generation solutions. Gut Microbes 12:1799654. doi: 10.1080/19490976.2020.1799654 32772817 PMC7524338

[B85] Wang W. Wang C. Dong Y. Yang F. Xu Y. (2025). Aneuploidy enables adaptation to brefeldin A in *Candida albicans*. Front. Cell. Infect. Microbiol. 15:1562726. doi: 10.3389/fcimb.2025.1562726 40357392 PMC12066683

[B86] Wang Z. Qin K. Camacho M. V. Cali I. Yuan J. Shen P. . (2021). Generation of human chronic wasting disease in transgenic mice. Acta Neuropathol. Commun. 9:158. doi: 10.1186/s40478-021-01262-y 34565488 PMC8474769

[B87] Wei D. W. Song Y. Li Y. Zhang G. Chen Q. Wu L. . (2025). Insertion sequences accelerate genomic convergence of multidrug resistance and hypervirulence in *Klebsiella pneumoniae* via capsular phase variation. Genome Med. 17:45. doi: 10.1186/s13073-025-01474-0 40329368 PMC12057282

[B88] West P. T. Peters S. L. Olm M. R. Yu F. B. Gause H. Lou Y. C. . (2021). Genetic and behavioral adaptation of *Candida parapsilosis* to the microbiome of hospitalized infants revealed by *in situ* genomics, transcriptomics, and proteomics. Microbiome 9:142. doi: 10.1186/s40168-021-01085-y 34154658 PMC8215838

[B89] Wu P. Wang Q. Yang Q. Feng X. Liu X. Sun H. . (2023). A novel role of the two-component system response regulator UvrY in enterohemorrhagic *Escherichia coli* O157:H7 pathogenicity regulation. Int. J. Mol. Sci. 24:2297. doi: 10.3390/ijms24032297 36768620 PMC9916836

[B90] Yap P. S. Cheng W. H. Chang S. K. Lim S. E. Lai K. S. (2022). MgrB mutations and altered cell permeability in colistin resistance in *Klebsiella pneumoniae*. Cells 11:2995. doi: 10.3390/cells11192995 36230959 PMC9564205

[B91] Zarsky V. Karnkowska A. Boscaro V. Trznadel M. Whelan T. A. Hiltunen-Thoren M. . (2023). Contrasting outcomes of genome reduction in mikrocytids and microsporidians. BMC Biol. 21:137. doi: 10.1186/s12915-023-01635-w 37280585 PMC10245619

[B92] Zhang H. Zou C. Peng O. Ashraf U. Xu Q. Gong L. . (2023). Global dynamics of porcine enteric coronavirus PEDV epidemiology, evolution, and transmission. Mol. Biol. Evol. 40:msad052. doi: 10.1093/molbev/msad052 36869744 PMC10027654

[B93] Zhang J. Xu Y. Wang M. Li X. Liu Z. Kuang D. . (2023). Mobilizable plasmids drive the spread of antimicrobial resistance genes and virulence genes in *Klebsiella pneumoniae*. Genome Med. 15:106. doi: 10.1186/s13073-023-01260-w 38041146 PMC10691111

[B94] Zhang M. Fan S. Liang M. Wu R. Tian J. Xian J. . (2024). A panoramic view of the molecular epidemiology, evolution, and cross-species transmission of rosaviruses. Vet. Res. 55:145. doi: 10.1186/s13567-024-01399-3 39516900 PMC11545274

[B95] Zhang Y. Qiu Y. Xue X. Zhang M. Sun J. Li X. . (2021). Transcriptional regulation of the virulence genes and the biofilm formation associated operons in Vibrio parahaemolyticus. Gut Pathog. 13:15. doi: 10.1186/s13099-021-00410-y 33653369 PMC7923509

[B96] Zhou J. Ma H. Zhang L. (2023). Mechanisms of virulence reprogramming in bacterial pathogens. Annu. Rev. Microbiol. 77, 561–581. doi: 10.1146/annurev-micro-032521-025954 37406345

[B97] Zoppo M. Poma N. Di Luca M. Bottai D. Tavanti A. (2021). Genetic manipulation as a tool to unravel *Candida parapsilosis* species complex virulence and drug resistance: state of the art. J. Fungi 7:459. doi: 10.3390/jof7060459 34200514 PMC8228522

